# Exploration of the potential neurotransmitter or neuromodulator-like properties of harmine: evidence from synthesis to synaptic modulation

**DOI:** 10.3389/fphar.2025.1588105

**Published:** 2025-07-07

**Authors:** Zhejun Xie, Ning Cao, Manlin Li, Hanxue Wang, Huida Guan, Xuemei Cheng, Changhong Wang

**Affiliations:** ^1^ The MOE Key Laboratory for Standardization of Chinese Medicines, Shanghai Key Laboratory of Compound Chinese Medicines, Institute of Chinese Materia Medica, Shanghai University of Traditional Chinese Medicine, Shanghai, China; ^2^ Shanghai TCM-Integrated Hospital, Shanghai University of Traditional Chinese Medicine, Shanghai, China

**Keywords:** harmine, neurotransmitter, neuromodulator, adipocyte plasma membrane-associated protein, central nervous system, receptor interaction

## Abstract

**Background:**

The discovery of new neurotransmitters is crucial for the in-depth understanding of neural signal transmission, neurological disorders, and relevant treatment strategies. Emerging evidence has indicated that harmine is an important endogenous compound, and its level is closely related to different physiological and disease states. Inspired by this, we propose a hypothesis that harmine may be a potential neurotransmitter or neuromodulator and display neurotransmitter or neuromodulator-like properties. This study aims to explore the potential properties of harmine as a neurotransmitter or neuromodulator according to the essential criteria for neurotransmitters and neuromodulators.

**Methods:**

Candidate proteins for the biosynthesis of harmine were searched by local BLAST. The target protein was then recombinantly expressed, purified, and functionally validated. Subsequently, the release, metabolism, and uptake pathways of harmine were investigated using rat brain synaptosomes and primary neural cells through mass spectrometry analysis. A human proteome microarray was employed to screen for harmine-binding receptors, followed by experimental validation.

**Results:**

Adipocyte plasma membrane-associated protein isoform X1 (APMAP-X1) could effectively catalyze the Pictet-Spengler reaction in mammals to generate tetrahydroharmine, which was subsequently oxidized by myeloperoxidase (MPO) to produce harmine. Moreover, harmine could be metabolized, taken up, and released within the synaptic cleft, fulfilling the conditions for clearance and release within the synaptic cleft. Harmine also regulated the expression of neurotransmitter transporters, implying its potential neuromodulatory properties. G protein-coupled receptor 85 (GPR85) and chloride intracellular channel 2 (CLIC2) were receptors targeted by harmine in the central nervous system. Functional verification results confirmed that harmine exerted an inhibitory effect on the GPR85 and could induce cellular depolarization.

**Conclusion:**

Current findings provide preliminary evidence that harmine may exhibit neurotransmitter-like properties in certain respects and support its role as an endogenous neuromodulator. However, direct evidence supporting harmine as a neurotransmitter remains limited. Further studies are needed to clarify the precise mechanisms of harmine in neurotransmission. Our study provides a new perspective for researchers on exploring novel endogenous molecules and their significance in neurophysiology.

## 1 Introduction

Neurotransmitters, serving as chemical messengers that transmit information between neurons and target cells, play a crucial role in the nervous system (Bian et al., 2023). Dysfunction of neurotransmitters is often closely associated with the development and progression of various neurological disorders (Nimgampalle et al., 2023). Similarly, neuromodulators, another class of important chemical substances in the central nervous system (CNS), can influence the activity of neurons by regulating the release and actions of neurotransmitters (Picciotto et al., 2012). Therefore, the discovery and study of new neurotransmitters is of great importance for a comprehensive understanding of the functions of the nervous system and the mechanisms of related diseases. With the continuous advancement in research on neurodegenerative diseases, harmine is gradually being recognized as an important endogenous bioactive compound (Louis et al., 2010; Cao et al., 2021).

β-Carboline alkaloids (β-CAs), including harmine, harmaline, and harmane, have been found to possess various neuropharmacological effects (Li et al., 2017). As inhibitors of monoamine oxidase and cholinesterase, they are considered to have potential therapeutic effects on various neuropsychiatric disorders such as Alzheimer’s disease (AD), depression, anxiety and some others (Hilber and Chapillon, 2005; Farzin and Mansouri, 2006; Zheng et al., 2009; Liu et al., 2014; He et al., 2015; Jiang et al., 2015; Liu et al., 2017; Li et al., 2018; Nasehi et al., 2018; Ebrahimi-Ghiri et al., 2019; Jiang et al., 2019; Nasehi et al., 2019; Mosaffa et al., 2021). It has been proven that some β-CAs can bind to multiple receptors, such as γ-aminobutyric acid receptors, benzodiazepine receptors, and 5-hydroxytryptamine receptors, but the mechanisms are not yet fully understood (Farzin and Mansouri, 2006; Nasehi et al., 2017; Ebrahimi-Ghiri et al., 2019).

β-CAs are widely present in plants and foodstuffs such as *Peganum harmala*, beverages, cooked meats and tobacco (Cao and Wang, 2021; Xie et al., 2021). Recently, these β-CAs have also been found in mammals, and their levels were closely associated with neurological disorders (Louis et al., 2010; Cao et al., 2021). Our studies further demonstrated the natural presence of harmine in human and rat plasma (Li et al., 2016; Cao et al., 2021). It could be detected in the plasma of newborn pup rats, absent of any external consumption. Its presence persisted throughout rats’ growth, but gradually decreased with aging, which is consistent with many classical neurotransmitters (Cao et al., 2021). These results indicated that harmine is an endogenous compound in mammals, and apart from exogenous intake, endogenous synthesis may be another important source of harmine. It prompts a crucial question: how is harmine biosynthesized in mammals? Furthermore, given its widespread distribution in tissues and fluids, alongside its various pharmacological activities, could harmine function as a neurotransmitter or neuromodulator? To address this pivotal scientific challenge, our research proceeded based on the essential criteria for neurotransmitters and neuromodulators (Bian et al., 2023; Picciotto et al., 2012): 1) The neurotransmitter must be synthesized within the presynaptic neurons, which requires the presence of precursors and associated enzymatic systems for its biosynthesis; 2) Upon arrival of the nerve impulse at the axon terminal, the neurotransmitter must be released from synaptic vesicles into the synaptic cleft; 3) There must be a mechanism to rapidly reduce the neurotransmitter concentration in the synaptic cleft, which can occur through enzymatic degradation, reuptake, or diffusion; 4) Once released into the synaptic cleft, the neurotransmitter must bind to receptors on the postsynaptic membrane, leading to a physiological effect. By contrast, the neuromodulator should affect neural signaling by influencing neurotransmitter levels and actions as well as the excitability of postsynaptic cells. Our investigations were conducted from multiple perspectives, including synthesis, clearance mechanisms, and receptor interactions, in attempt to elucidate whether harmine possesses the properties of a neurotransmitter or neuromodulator.

## 2 Materials and methods

### 2.1 Materials

Harmine (purity: >98%, CAS: 442-51-3) and harmol (purity: 98%, CAS: 487-03-6) were purchased from TCI (Japan). D-biotin (purity: 99%, CAS: 58-85-5) was purchased from J&K Scientific (China). Tryptamine (CAS: 61-54-1), secologanin (CAS: 19351-63-4), glucose-6-phosphatase (G-6-P), glucose-6-phosphate dehydrogenase (G-6-P-H), nicotinamide adenine dinucleotide phosphate (NADPH), alamethicin (ALA), uridine diphosphate glucuronic acid (UDPGA), and glucose were obtained from Merck (Germany). 2-(7-Azabenzotriazol-1-yl)-N,N,N′,N′-tetramethyluronium hexafluorophosphate (HATU, BR, cat: 39204934), 4-dimethylaminopyridine (DMAP, AR, cat: 30198415), and N,N-dimethylformamide (DMF, AR, cat: 81007718) were obtained from Sinopharm Chemical Reagent Co., Ltd. (China). Deuterated dimethyl sulfoxide (99.8 atom%D + 0.03% v/v TMS) was purchased from manalab^®^ (China). Deuterated pyridine (99.5 atom%D + 0.05% v/v TMS) was obtained from Cambridge Isotope Laboratories, Inc (USA). DMEM (cat: 11995065), FBS (cat: 1913769) and PBS (cat: 10010023) were purchased from Gibco (USA). SteadyPure RNA Extraction Kit (cat: AG21024), AG RNAex Pro reagent (cat: AG21101), Evo M-MLV RT Premix for qPCR (cat: AG11706), and SYBR^®^ Green Premix Pro Taq HS qPCR Kit (Rox Pus) (cat: AG11718) were purchased from Accurate Biology (China). Tryptone (FMB Grade), yeast extract (FMB Grade), agar powder (FMB Grade), and ampicillin sodium (USP Grade) were purchased from Sangon Biotech (Shanghai) Co., Ltd. (China). Plasmid extraction kits (cat: D6943-01) were obtained from Omega (USA). DNA transfection reagent was purchased from NEOFECT (China). Recombinant anti-CREB (phosphor S133) antibody (cat: ab32096) and GAPDH antibody (cat: ab181602) were obtained from Abcam (UK). BCA protein assay kits, sample loading buffer, and goat anti-rabbit HRP antibody (cat: 33101ES60) were purchased from Yeasen Biotechnology Co., Ltd. (China). Chromatographic grade acetonitrile, methanol, and formic acid were purchased from Thermo Fisher Scientific (USA). Ultra-pure water was filtered by a Milli-Q Academic System (Millipore, USA). The animal experiments were approved by the Animal Care and Use Committee of Shanghai University of Traditional Chinese Medicine (Approval NO. PZSHUTCM190912018, NO. PZSHUTCM2501090001). The animals were housed in an environment-controlled room (temperature: 22°C–25°C, humidity: 40%–60%, a 12 h light-dark cycle). Measures were taken to minimize animal suffering, including anesthesia during procedures, close monitoring, and euthanasia.

### 2.2 Identification of the key protein and functional verification

#### 2.2.1 Plasmid recombination, recombinant protein expression and purification

Inspired by studies discovering that β-carboline alkaloids (β-CAs) in plants are generated via the Pictet-Spengler reaction (P-S reaction) (Cao and Wang, 2021), our investigation began with the identification of key proteins involved in the synthesis of harmine in mammals, aiming to explore the potential for its endogenous synthesis. NCBI protein database and Local BLAST were employed using strictosidine synthase (CAA71255.1) as a template for candidate protein screening. Among them, the highest matching protein was adipocyte plasma membrane-associated protein isoform X1 (APMAP-X1), which was selected for further study.

Plasmid construction: Coden-optimized APMAP-X1 nucleotide sequence (XP_006235277.1) was synthesized by Nanjing Genscript Biotechnology Co., Ltd. Transmembrane region of APMAP-X1 was predicated using website http://www.cbs.dtu.dk/services/TMHMM/. The result showed that amino acids 1-36 are located intracellularly, amino acids 37-59 constitute the transmembrane region, and amino acids 60-415 are extracellular. Therefore, amino acids 1-59 were removed to enhance solubility and improve expression levels.

The NdeI and XhoI restriction enzyme sites were chosen to perform enzymatic digestion of the pET28a vector. Subsequently, the digested vector and PCR product (1:1) were ligated by infusion enzyme, and the products were transformed into DH5α competent cells by heat-shock method. The expression plasmid with correct sequence was named as pET28a-APMAP-X1.

The EcoRI and XbaI restriction enzyme sites were chosen to perform enzymatic digestion of the pGAPZB vector and PCR products. The digested vector and PCR product (1:3 to 1:10) were ligated by T4 DNA Ligase, and the products were transformed into DH5α competent cells by heat-shock method. The expressed plasmid with correct sequence was named as pGAPZB-APMAP-X1.

Recombinant protein expression: The constructed plasmid pET28a-APMAP-X1 was transformed into *E. coli* BL21 (DE3) competent cell. The positive strains were obtained through kanamycin resistance screening on Luria-Bertani (LB) plate, followed by amplified cultivation. After induction with isopropyl-β-D-1-thiogalactopyranoside (IPTG, 1 mM), the strains were collected. The expression of recombinant protein was identified by sodium dodecyl sulfate-polyacrylamide gel electrophoresis (SDS-PAGE), followed by Coomassie Brilliant Blue R-250 staining. Since the target protein was mainly concentrated in the pellet, the inclusion body refolding method was employed to solubilize the target protein for activity verification.

The constructed plasmid pGAPZB-APMAP-X1 was transformed into *Peganum pastoris* GS115 competent cell by electrotransformation. Prior to transformation, DNA plasmids were linearized using DraⅡ restriction enzyme. The positive strains were obtained through zeocin resistance screening on yeast extract peptone dextrose (YPD) plate. Subsequently, PCR amplification and agarose gel electrophoresis were performed, and the successfully transformed strains were named as APMAP-X1-pGAPZB-GS115. The strains were collected, and the expression of recombinant protein was identified by SDS-PAGE, followed by Coomassie brilliant blue R-250 staining.

#### 2.2.2 Catalytic activity validation of purified protein *in vitro*

Subsequently, the catalytic activity of purified APMAP was further evaluated through *in vitro* incubation and strictosidine analysis using secologanin (SEC) and tryptamine (TRY) as substrates. The incubation system for activity verification was shown below: cofactor: ALA (50 μg/mL), G-6-P (10 mM), G-6-P-H (1 unit/mL), NADPH (1 mM), UDPGA (5 mM) and KCl (4 mM), purified protein (1 mg/mL), substrates: SEC (600 μM) and TRY (600 μM). The samples were incubated at 37°C for 1 h. After incubation, 1 mL water-saturated butanol was added and then vortexed for 2 min to terminate the reaction. Then the mixture was centrifuged at 13,000 × *g* for 10 min, and the supernatant was dried by nitrogen blowing. After re-dissolving, 5 μL aliquot of supernatant was injected into the UHPLC-QTOF-MS/MS system for analysis.

The products were detected using a triple TOF™ 5600^+^ system (AB Sciex, CA, USA) coupled with a Shimadzu LC-30 AD UHPLC system (Shimadzu, Kyoto, Japan). The mass spectrometer was operated in the negative ion mode with capillary voltage of −4,500 V. The turbo spray temperature was 550°C, and the declustering potential (DP) was 60 V. The collision energy (CE) was set to 35 eV, and the collision energy spread (CES) was set to 15 eV. The flow rates of nebulizer gas (gas 1), heater gas (gas 2), and curtain gas were set to 55 psi, 55 psi, and 35 L/min, respectively. The full scan range was covered from m/z 50 to m/z 1,200. The UPLC BEH C_18_ column (100 × 2.1 mm, 1.7 μm, Waters, USA) was employed. The mobile phase consisted of 0.1% formic acid in water (A) and acetonitrile (B) with the gradient elution: 0-1.5 min, 2%-20% B, 1.5-4 min, 20%-65% B, 4-11 min, 65%-95% B, 11-15 min, 95% B, 15-20 min, 95%–2% B. The column temperature was set to 45°C, and the flow rate was set to 0.4 mL/min.

#### 2.2.3 Substrates screening for the synthesis of harmine *in vitro*

Given that harmine is a β-carboline alkaloid and the β-carboline scaffold can be efficiently synthesized via the P-S reaction, various amines and aldehydes were selected as substrates in accordance with its structural characteristics, including formaldehyde (FA), acetaldehyde (AA), butyraldehyde (BA), iso-valeraldehyde (iVA), TRY, 5-hydroxytryptamine (5-HT), and 6-methoxy-tryptamine (MOT). The purified APMAP, substrates and other cofactors were co-incubated at 37°C for 20 min. After incubation, 500 μL acetonitrile was added and then vortexed for 2 min to terminate the reaction. Then the mixture was centrifuged at 13,000 × *g* for 10 min and the supernatant was dried by nitrogen blowing. After re-dissolving by 10% acetonitrile-water, 5 μL aliquot of supernatant was injected into the UHPLC-QTOF-MS/MS system for analysis.

Building upon the P-S reaction catalyzed by APMAP, myeloperoxidase (MPO) was introduced to catalyze oxidation reaction. The mixtures were co-incubated at 37°C for 1 h. 5 μL aliquot of supernatant was injected into the UHPLC-QTOF-MS/MS system for analysis. The retention time and fragment ion of products were compared with those of harmine standard.

The products were detected using a triple TOF™ 5600^+^ system equipped with ESI mode and coupled to a Shimadzu 30 A UHPLC system. The mass spectrometer was operated in the positive mode with capillary voltages of 5500 V. The turbo spray temperature was 550°C, and the DP, CE and CES was set to 60 V, 25 eV, and 15 eV, respectively. The flow rates of nebulizer gas (gas 1), heater gas (gas 2), and curtain gas were set to 35 L/min, respectively. The full scan range was covered from mass m/z 50 to m/z 500. The samples were separated using UPLC BEH C_18_ column (2.1 × 100 mm, 1.7 µm). The mobile phase consisted of 0.1% formic acid in water (A) and acetonitrile (B) with the gradient elution: 0–2min, 10% B, 2–5 min, 10%–30% B, 5–8 min, 30%–90% B, 8–10 min, 90% B, 10.1–11 min, 10% B. The column temperature was set to 45°C, the flow rate was set to 0.3 mL/min, and the injection volume was 5 μL.

### 2.3 Metabolism of harmine in synaptosomes

The rat brain synaptosomes were prepared by differential centrifugation according to the protocol of Gobbi et al. (2002). The metabolic incubation system was shown below: cofactor: Tris-HCl (50 mM), G-6-P (10 mM), G-6-P-H (1 unit/mL), NADPH (1 mM), and MgCl2 (4 mM), brain synaptosome (1.3 mg/mL), substrate: harmine (200 μM). A no-substrate control group was included in this experiment. The samples were incubated at 37°C for 1 h. After incubation, 500 μL ice-cold acetonitrile was added and then vortexed for 1 min to terminate the reaction. Then the mixture was centrifuged at 13,000 × g for 10 min, and the supernatant was dried by nitrogen blowing. After re-dissolving, 5 μL aliquot of supernatant was injected into the UHPLC-QTOF-MS/MS system for analysis.

### 2.4 Uptake and release of harmine

The uptake and release assays were performed following the protocols described by Gobbi et al. (2002) Abu Ghazaleh et al. (2015). In the synaptosome uptake experiment, the concentration of harmine was set to 10 μM, and was incubated with synaptosomes at 37°C for 0, 5, 10, 20, 40, and 60 min. After incubation, the mixture was centrifuged at 15,000 × *g* for 15 min at 4°C. The pellet was washed twice with uptake buffer and was finally resuspended in 50 μL buffer. 250 μL of acetonitrile containing internal standard was added, and the mixture was vortexed for 3 min. After centrifugation at 13,000 × *g* for 10 min at 4°C, the supernatant was collected and dried under a nitrogen stream. After re-dissolving, 5 μL aliquot of supernatant was injected into the UHPLC-MS/MS system for analysis.

In the synaptosome release experiment, harmine was fixed at a final concentration of 10 μM and incubated with brain synaptosomes at 37°C for 20 min. The samples were then centrifuged at 15,000 × *g* for 15 min at 4°C. After centrifugation, the supernatant was removed and the synaptosomes were washed twice with uptake buffer. Subsequently, 100 μL high K^+^ release buffer was added, and samples were collected at 0, 10, 20, 40, and 60 min to measure the release amount. Sample processing and detection methods were carried out as described previously.

In the cell uptake experiment, primary neurons and astrocytes were seeded in 6-well plates. Prior to the experiment, the culture medium was discarded, and the cells were washed twice with pre-warmed uptake buffer. The cells were then treated with harmine (5 μM) and incubated at 37°C for 0, 5, 10, 20, 30, 40, 60, and 120 min. Finally, the cells were collected for harmine content analysis using the established UHPLC-MS/MS method. Different concentrations of harmine (1, 5, 10 μM) on the uptake of harmine by primary neurons and astrocytes were also assessed.

### 2.5 Synthesis, purification, and characterization of the harmine-biotin complex

**FIGURE 1:**
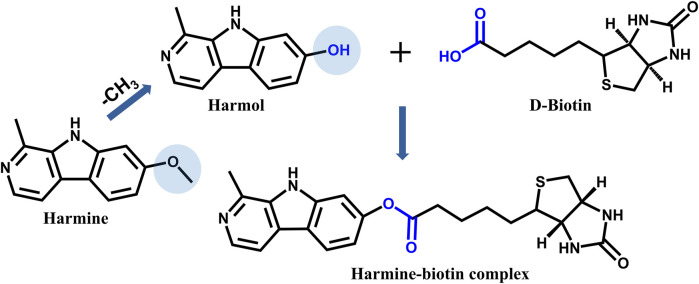
The schematic representation of the chemical synthesis of harmine-biotin complex.

To further investigate the receptors interacting with harmine, the human proteome microarray was employed for receptor screening. As a preliminary step, harmine needed to be biotinylated to enable its detection and analysis. After evaluation, it was decided to conjugate D-biotin at the methoxy group site on the C-7 position of harmine. Since harmine itself is challenging to directly conjugate with biotin, its precursor (harmol) was selected as the starting material for synthesis. Biotin (1.1 eq), HATU (2 eq), and DMAP (1 eq) were dissolved in DMF and stirred for 20 min. Subsequently, harmol (1 eq) was added to the reaction system, and the mixture was stirred at room temperature overnight (Figure 1).

The synthesized product was separated and purified using a preparative liquid chromatography system. A SHISEIDO CAPCELL PAK C_18_ (2.1 × 100 mm, 1.8 μm) was used for separation. Chromatography condition 1: The mobile phase consisted of water (A) and methanol (B) at a flow rate of 16 mL/min. The optimized gradient elution conditions were as follows: 0–10.00 min, 20%–25% B; 10.10–20.00 min, 40%–50% B; 20.10–35.00 min, 80% B. Chromatography condition 2: The mobile phase consisted of water (A) and acetonitrile (B) at a flow rate of 10 mL/min. The optimized gradient elution conditions were as follows: 0–8.00 min, 30% B; 8.10–30.00 min, 50% B.

The purity and structure of the purified product were confirmed by high-performance liquid chromatography (HPLC), mass spectrometry (MS), and nuclear magnetic resonance (NMR) spectroscopy. HPLC analysis was conducted using a Diamonsil Plus C_18_ column (5 μm, 250 × 4.6 mm). The mobile phase consisted of 0.1% formic acid in water (A) and acetonitrile (B). The gradient elution condition was as follows: 0–60.00 min, 5%–95% B. The flow rate was set at 1 mL/min. UHPLC-QTOF-MS/MS analysis was performed using an ACQUITY UPLC BEH C_18_ column (1.7 μm, 2.1 × 100 mm). The mobile phase consisted of 0.1% formic acid in water (A) and acetonitrile (B). The flow rate was 0.4 mL/min with a column temperature of 30°C. The optimized gradient elution conditions were as follows: 0.50–1.50 min, 2%–20% B; 4.00–11.00 min, 65%–95% B; 15.00–15.10 min, 95%–2% B; 20.00 min, stop. The mass spectrometer was operated in the positive ion full scan mode. The conditions were set as follows: source temperature: 550°C, capillary voltage: 5500 V, declustering potential: 80 V, collision energy: 35 eV, collision energy spread: 15 eV, nebulizer gas: 55 psi, heater gas: 55 psi, and curtain gas: 35 L/min.

### 2.6 Human proteome microarray

The HuProt™ 20K Human Proteome Microarrays were employed to identify receptors interacting with harmine. The procedure was carried out according to the standard protocol provided by CDI Microarray Company. The microarrays were scanned using the GenePix 4000B scanner at 635 nm wavelength. Data analysis was conducted using GenePix™ Pro v6.0 software. D-biotin was used as a control to exclude signals caused by biotin labeling. The median and standard deviation (SD) of signal intensities (I) were calculated for all sites. Z-scores were computed to normalize data at each site. Typically, Z-score >2.8 is generally considered as the detection threshold, indicating that the signal is significantly higher than the background level. The mean of intensity (I Mean) from two replicates for each protein was determined, generating intergroup ratios (I Mean_Ratio) for all proteins. Proteins with I Mean_Ratio ≥1.4 were the significantly specific proteins detected in the experimental group.

### 2.7 Cell culture and treatment

SH-SY5Y cells were cultured in DMEM containing 10% FBS and 1% penicillin-streptomycin at 37°C and 5% CO_2_. Cells were seeded in 6-well plates and were allowed to reach 70%–80% confluence before transfection. pLV3-CMV-GPR85 (human)-3×FLAG-CopGFP-Puro plasmid was transfected into cells using Neofect DNA transfection reagent. After transfection, cells were incubated with medium with or without harmine. Finally, cells were collected for Western blotting analysis.

### 2.8 Western blotting

Cells were lysed in RIPA buffer supplemented with protease and phosphatase inhibitors. Protein concentration was determined using a BCA protein assay kit. 15 μg protein were separated by 10% SDS-PAGE and transferred onto PVDF membranes. The membranes were incubated with primary antibodies against p-CREB, followed by incubation with HRP-conjugated secondary antibodies. The image was captured by Tanon 5200 imaging system.

### 2.9 Membrane potential detection

SH-SY5Y cells were seeded in 96-well plates and transfected with pLV3-CMV-CLIC2(human)-3×FLAG-CopGFP-Puro plasmid for 48 h prior to membrane potential detection. The cells were equilibrated with membrane potential buffer for 30 min prior to the experiment. Subsequently, an equal volume of 2×DiSBAC2(3) probe dye was added to each well and incubated with the cells for 75 min. Membrane potential was measured by detecting fluorescence intensity at Ex/Em = 540/590 nm using a microplate reader. Baseline measurements were taken prior to harmine treatment, after which various concentrations of harmine (1, 5, 10 μM) were added, and cells treated with membrane potential buffer served as the negative control. Fluorescence intensity changes were monitored at different time points.

### 2.10 Quantitative real-time PCR

Total RNA from primary neural cells was isolated by SteadyPure RNA Extraction Kit and was reverse-transcribed into cDNA by Evo M-MLV RT Premix. RT-PCR was performed using SYBR^®^ Green Premix Pro Taq HS qPCR Kit by real-time fluorescence quantitative PCR instrument. The relative expression of target genes was analyzed by the 2^−ΔΔCT^ method. β-Actin was used as an internal control. The sequences of real-time PCR primers were shown in Table 1.

**TABLE 1:** The list of primers for RT-qPCR.

Target gene	Sequences (5′-3′)
DAT1	F: TCT​ACC​GAC​TCT​GTG​AGG​CAT​CTG
	R: AGA​AGG​CAA​TCA​GCA​CTC​CAA​ACC
SERT	F: GCC​ACC​TTC​CCA​TAC​ATT​GTC​CTC
	R: TCT​GAG​CGG​CGG​CAT​CTA​CC
NET	F: GAT​TGA​GGA​TGT​CGC​CAC​TGA​AGG
	R: GCC​AGG​AGC​ATC​AGG​AAG​AAC​AG
GLYT1	F: GGT​TGT​CTT​CCT​CTG​CCT​CAT​TCG
	R: CCA​GGG​TCA​CTC​CAC​GAA​CAA​AC
GAT1	F: CGG​CTT​CGT​CAT​CTT​CTC​CAT​CG
	R: GGT​ACG​CCA​AGA​ATG​CCA​GTC​C
GAT3	F: GGT​GGA​GTT​CGT​GTT​GAG​CGT​AG
	R: GCC​GTT​CTT​GTA​GCA​CAG​GTA​GG
EAAT1	F: AGC​CAC​AGC​CGC​AAG​CAT​TG
	R: GCC​CAC​AGA​TGT​CAG​CAC​GAT​G
EAAT2	F: GAG​GAG​CCA​AAG​CAC​CGA​AAC​C
	R: CGA​AGC​AGC​CCG​CCA​CAT​AC
EAAT3	F: GCA​TCG​TGG​TAG​GAG​TCT​TGG​TTC
	R: CGA​GCT​TCA​GCA​TCC​GCA​TCA​G
ACTB	F: ACT​GCC​GCA​TCC​TCT​TCC​TC
	R: GAA​CCG​CTC​ATT​GCC​GAT​AGT​G

### 2.11 Statistical analysis

The results were expressed as mean ± SD. Statistical analysis was performed using GraphPad Prism 8 software. The data between the two groups were analyzed using t-test. one-way ANOVA was used to compare the data between multiple groups. Shapiro-Wilk tests were used to evaluate the normality of data distribution, and Bartlett’s test was applied to assess the homogeneity of variances. If data met the assumptions of normality and homogeneity, unpaired t-test or one-way ANOVA was used. If data did not meet the assumption of normality, non-parametric Mann-Whitney test or non-parametric Kruskal–Wallis test was performed instead. Dunnett’s post hoc multiple comparison tests were performed to identify specific group differences. ^*^p < 0.05, ^**^p < 0.01, ^***^p < 0.001, ^****^p < 0.0001 were considered statistically significant.

## 3 Results

### 3.1 Discovery of key protein catalyzing P-S reaction in mammals

#### 3.1.1 The expression and purification of APMAP-X1 in *E. coli* BL21 (DE3) and *P. pastoris* GS115

The coding region of APMAP-X1 gene was obtained by the total gene synthesis technology. The constructed gene was amplified by PCR, and the PCR products (pET28a-APMAP-X1 and pGAPZB-APMAP-X1) were identified by gel electrophoresis.

**FIGURE 2:**
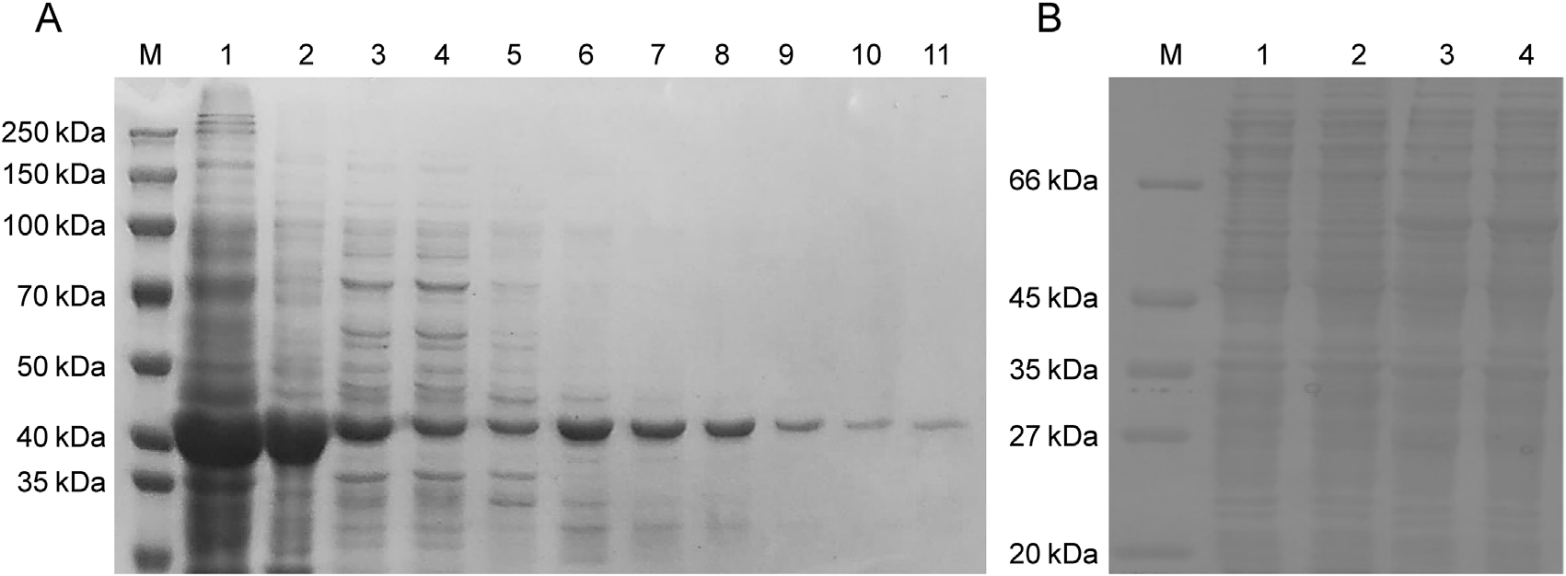
Analysis of soluble APMAP-X1 protein in *E. coli* BL21 (DE3) and *P. pastoris* GS115 by SDS-PAGE. **(A)** Purification of recombinant APMAP-X1 in *E. coli* BL21 (DE3). Channel 1: the inclusion body, channel 2: the precipitate after the dissolution of inclusion body, channel 3: the inclusion body after dialysis renaturation, channel 4: the supernatant after adsorption on the Ni affinity column, channel 5: 50 mM imidazole eluent, channels 6, 7: 100 mM imidazole eluent, channels 8, 9: 250 mM imidazole eluent, channels 10, 11: 500 mM imidazole eluent. **(B)** Purification of recombinant APMAP-X1 in *P. pastoris* GS115. Channel 1: soluble protein in GS115/pGAPZB colony, Channel 2, 3, 4: soluble protein in the supernatant of GS115/pGAPZB-APMAP-X1 after 2, 4 and 6 days of induction.

The expression and purification of APMAP-X1 in *E. coli* BL21 (DE3): The constructed gene, with restriction site XhoⅠ and NdeⅠ, was amplified through PCR. A band near 1,000 bp was identified in the electrophoresis result of pET28a-APMAP-X1 (Supplementary Figure 1A), consistent with the expected size of the designed sequence (1,071 bp). In addition, the pET28a-APMAP-X1 was verified to have the correct sequence, indicating the successful construction of the recombinant plasmid. Subsequently, the expression of recombinant APMAP-X1 in induced BL21 (DE3)/pET28a-APMAP-X1 was identified through SDS-PAGE. A protein band with a molecular weight of approximately 45 kDa was observed in the induced BL21 (DE3)/pET28a-APMAP-X1 strain, whereas there was minimal protein expression in the uninduced strain (Supplementary Figure 1B), indicating successful expression of the recombinant APMAP-X1. As shown in Supplementary Figure 1C, almost all expressed recombinant protein was found in the pellet. Thus, the inclusion bodies in pellet were isolated and purified for further activity validation. As illustrated in Figure 2A, compared to channel 1 and 2, washing could remove some of the contaminating proteins. During the refolding process, a portion of the inclusion bodies re-precipitated, leading to a reduction in the target protein band in channel 3. The Ni^+^ affinity column did not fully adsorb the target protein and hence the target protein band was still visible in the eluate after adsorption (channel 4). The pure target protein was obtained from second half of 250 mM imidazole eluent.

The expression and purification of APMAP-X1 in *P. pastoris* GS115: The constructed gene, with restriction site EcoRⅠ and XbaⅠ, was amplified through PCR. The band identified in the electrophoresis result of PCR product was consistent with the expected size of the designed sequence (Supplementary Figure 2A). The sequence of pGAPZB-APMAP-X1 has been verified to be correct, indicating the successful construction of recombinant plasmid. The PCR analysis results confirmed the successful insertion of the APMAP-X1 gene into *P. pastoris* GS115 (Supplementary Figure 2B). Bands of 1,283 bp were amplified in positive recombinant strains, indicating the successful insertion of the APMAP-X1gene into the strains. Protein expression in positive recombinant strains (pellet) was shown in Supplementary Figure 2C. A protein band with a molecular weight of about 55 kDa was observed in the pellet of GS115/pGAPZB-APMAP-X1 strain, while there was minimal protein expression in the pellet of GS115/pGAPZB strain, indicating the successful expression of APMAP-X1. Similar protein brand was also observed in the supernatant (Figure 2B), and this soluble protein was used for further activity validation.

#### 3.1.2 Activity validation of APMAP-X1 *in vitro*

Subsequently, the catalytic activity of the purified APMAP-X1 for P-S reaction was assessed by determining the formation of the desired product (strictosidine) using SEC and TRY as substrates. UHPLC-MS/MS analysis showed that APMAP-X1 expressed in *E. coli* BL21 (DE3) and *P. pastoris* GS115 exhibited catalytic activity, with detectable strictosidine in the incubation product (Supplementary Figure 3). The retention time and fragment ions were consistent with those of strictosidine standard. This result suggested that the purified APMAP-X1 was an enzyme that catalyzed the P-S reaction in mammals.

#### 3.1.3 Substrates screening for the synthesis of harmine *in vitro*

**FIGURE 3:**
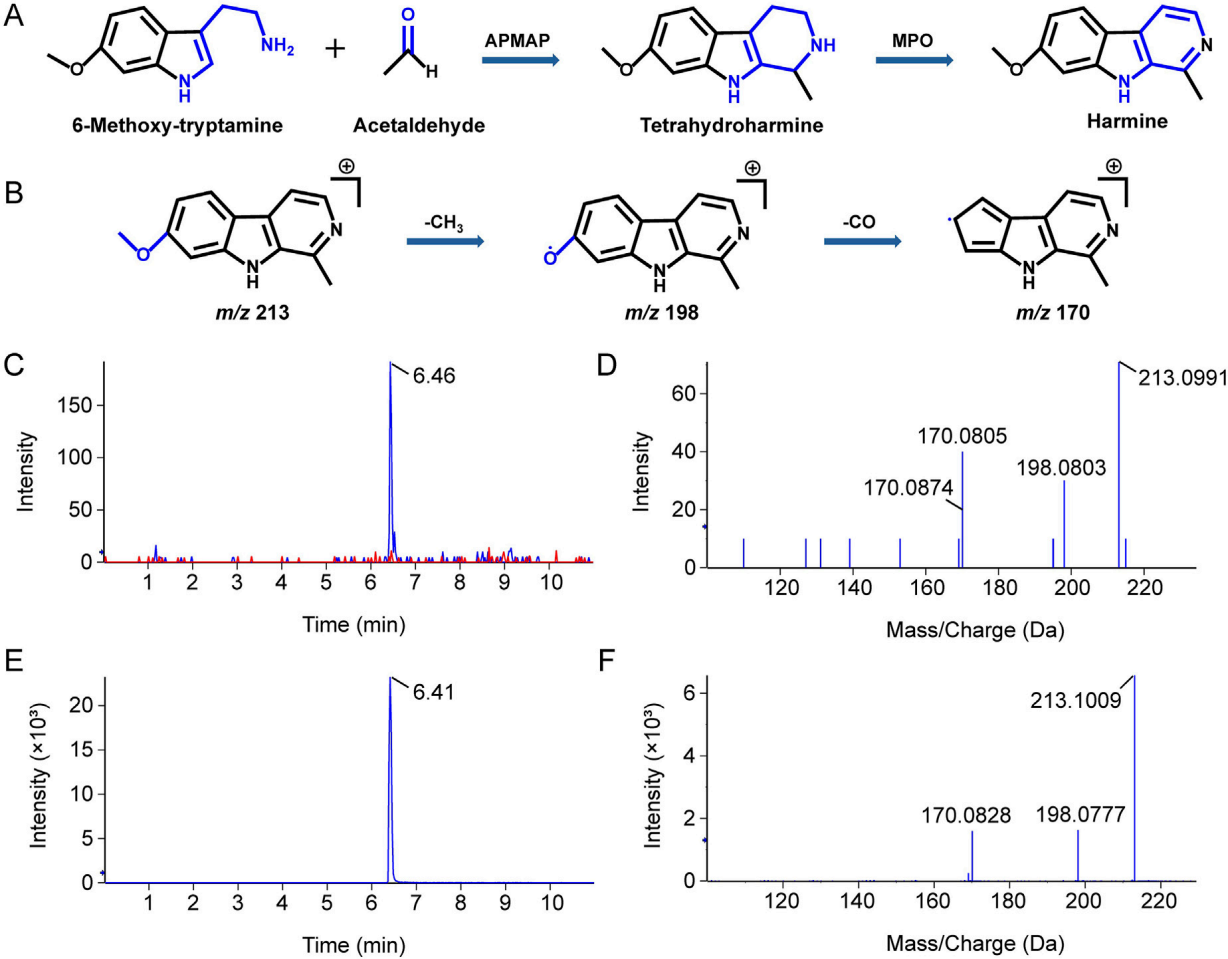
LC-MS/MS identification of products formed from the incubation of aldehyde and amine with APMAP and MPO. **(A)** Schematic representation of the reaction between 6-methoxytryptamine and acetaldehyde under the catalysis of APMAP and MPO. **(B)** Schematic illustration of the fragmentation of harmine. The XIC **(C)** and secondary ion fragments spectrum **(D)** of a reaction product with the molecular formula C_13_H_12_N_2_O. The XIC **(E)** and secondary ion fragments spectrum **(F)** of harmine standard.

To figure out the biosynthesis substrates of harmine, small molecule aldehydes and amines, including FA, AA, BA, iVA, TRY, 5-HT, and MOT, were applied for substrate specificity research. The UHPLC-QTOF-MS/MS results showed that MOT could react with AA to produce tetrahydroharmine under the catalysis of APMAP. However, the product of P-S reaction was tetrahydroharmine, hence further oxidation was needed to form harmine. Previous studies showed that heme peroxidases may be potential enzymes for facilitating subsequent oxidative step following the P-S reaction, thereby completing the biosynthesis of β-CAs (Herraiz et al., 2007; Herraiz and Galisteo, 2014; Wang et al., 2022). Therefore, MPO was added into the P-S reaction system for further oxidation. As shown in Figure 3, experimental group showed apparent product peak, with retention time and secondary fragments matching those of harmine. These results indicated that MOT and AA could generate harmine under the catalysis of APMAP and MPO.

### 3.2 Clearance and release pathway of harmine

#### 3.2.1 The metabolic pathway of harmine

To explore the clearance mechanisms of harmine from the synaptic cleft, its metabolism and uptake were investigated. Initially, the metabolic pathways of harmine were investigated using rat brain synaptosomes. As shown in Table 2, five metabolites of harmine were identified in the rat brain synaptosome incubation system, and the MS spectrums were shown in Supplementary Figure 4.

**TABLE 2:** Metabolites of harmine in rat brain synaptosomes.

Metabolites	RT (min)	Formula [M + H]^+^	Measured mass	Proposed metabolic pathways	Fragment ions
Harmine	6.25	C_13_H_13_N_2_O	213.1008		198.0768, 170.0819
M1	4.94	C_13_H_13_N_2_O_2_	229.0965	N-oxidation	214.0732, 186.0786, 157.0755
M2	6.46	C_13_H_13_N_2_O_2_	229.0953	Monohydroxylation	212.0926, 197.0700, 169.0747
M3	4.82	C_12_H_11_N_2_O	199.0857	Demethylation	184.0618, 171.0902, 131.0480
M4	6.24	C_12_H_11_N_2_O	199.0850	Demethylation	184.0628, 156.0670
M5	6.40	C_14_H_15_N_2_O	227.1164	Methylation	212.0933, 184.0984, 169.0752

The retention time (t_R_) of harmine was 6.25 min, and it exhibited a protonated molecular ion at *m/z* 213.1008 (C_13_H_13_N_2_O). M1 (t_R_ = 4.94 min) showed a [M + H]^+^ ion at *m/z* 229.0965 (C_13_H_13_N_2_O_2_), which was 16 Da higher than that of harmine. The MS/MS spectrum showed characteristic fragment ions at *m/z* 214.0732 (−15 Da, -CH_3_), 186.0786 (−28 Da, -C_2_H_3_O), and 157.0755 (−72 Da, -C_3_H_4_O). Therefore, M1 was identified as the N-oxide metabolite of harmine. M2 (t_R_ = 6.46 min) had a [M + H]^+^ ion at *m/z* 229.0953 (C_13_H_13_N_2_O_2_), which was 16 Da larger than that of harmine. The MS/MS spectrum showed characteristic fragment ions at *m/z* 212.0926 (−17 Da, -OH), 197.0700 (−32 Da, -CH_4_O), and 169.0747 (−60 Da, -C_2_H_4_O_2_). Therefore, M2 was identified as the hydroxylated metabolite of harmine. M3 (t_R_ = 4.82 min) was observed a [M + H]^+^ ion at *m/z* 199.0857 (C_12_H_11_N_2_O), which was 14 Da lower than that of harmine. The MS/MS spectrum showed characteristic fragment ions at *m/z* 184.0618 (−15 Da, -CH_3_), 171.0902 (−28 Da, -CO), and 131.0480 (−68 Da, -C_3_H_4_N). Therefore, M3 was identified as the demethylated metabolite of harmine. M4 (t_R_ = 6.24 min) exhibited a [M + H]^+^ ion at *m/z* 199.0850 (C_12_H_11_N_2_O), which was 14 Da less than that of harmine. The MS/MS spectrum showed characteristic fragment ions at *m/z* 184.0628 (−15 Da, -CH_3_) and 156.0670 (−43 Da, -C_2_H_3_O). Therefore, M4 was identified as the demethylated metabolite of harmine. M5 (t_R_ = 6.40 min) exhibited a [M + H]^+^ ion at *m/z* 227.1164 (C_14_H_15_N_2_O), which was 14 Da heavier than that of harmine. The MS/MS spectrum showed characteristic fragment ions at *m/z* 212.0933 (−15 Da, -CH_3_) 184.0984 (−43 Da, -C_2_H_3_O), and 169.0752 (−58 Da, -C_3_H_6_O). Therefore, M5 was identified as the methylated metabolite of harmine. These results indicated the presence of metabolic pathways of harmine in rat brain synaptosomes, primarily involving N-oxidation, hydroxylation, methylation, and demethylation.

#### 3.2.2 The uptake and release of harmine in brain synaptosomes and primary neural cells

**FIGURE 4:**
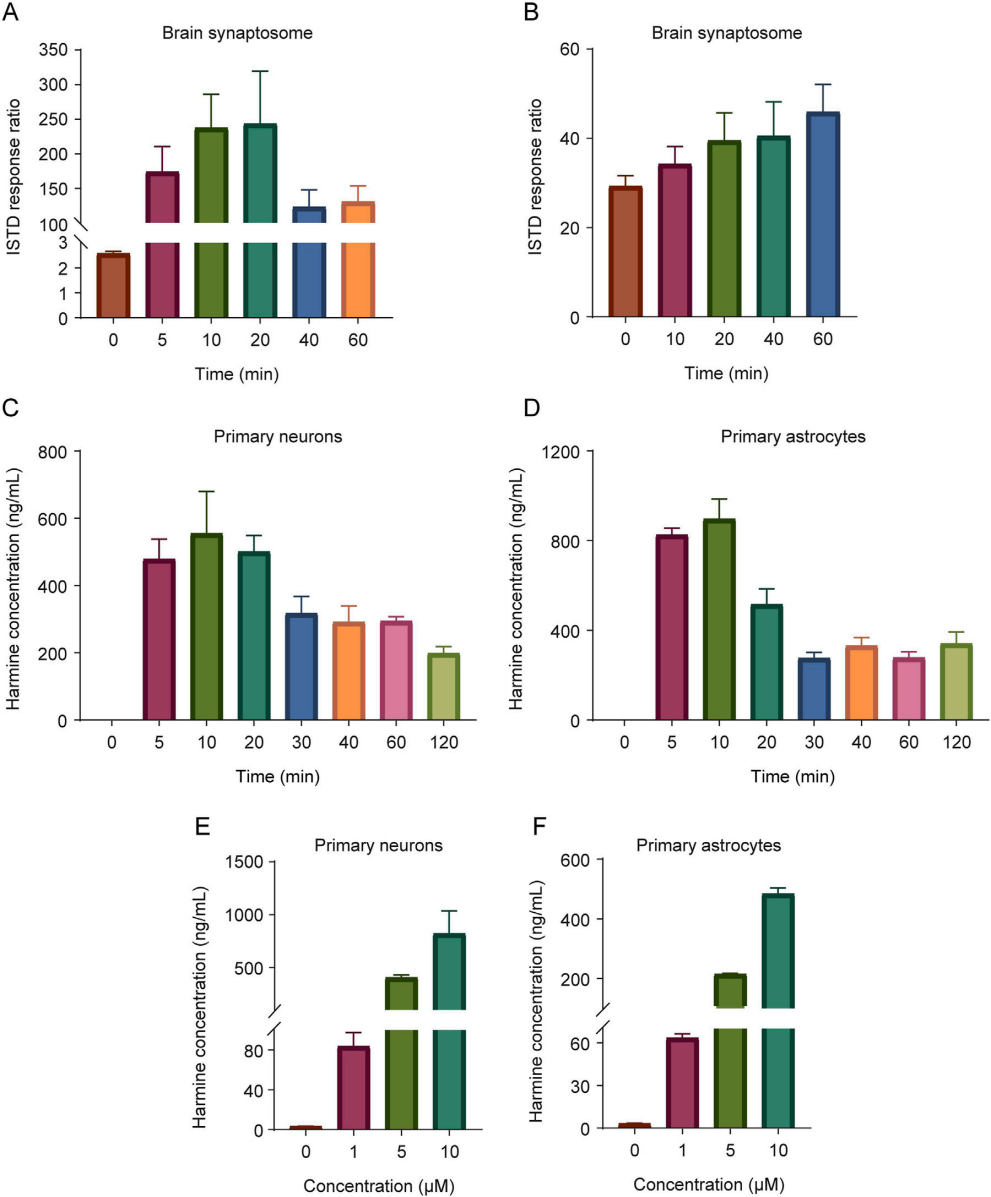
The uptake and release of harmine in the synaptosomes and primary neural cells. The uptake **(A)** and release **(B)** of harmine in brain synaptosomes. **(C)** The uptake of harmine in primary neurons. **(D)** The uptake of harmine in primary astrocytes. **(E)** The effect of harmine concentration on the uptake of harmine in primary neurons. **(F)** The effect of harmine concentration on the uptake of harmine in primary astrocytes. Data were presented as mean ± SD, n = 3/group.

To investigate the uptake and release of harmine, experiments were conducted in rat brain synaptosomes and primary neural cells. The results revealed that the uptake of harmine in brain synaptosomes increased initially and then declined over time, peaking at 20 min (Figure 4A). Meanwhile, the release amount of harmine increased continuously over time (Figure 4B). In neural cells, both primary neurons and astrocytes were able to take up harmine, with the highest uptake occurring around 10 min (Figures 4C,D). Additionally, the uptake of harmine in both cell types was dose-dependent (Figures 4E,F).

### 3.3 The effect of harmine on neurotransmitter transporters

**FIGURE 5:**
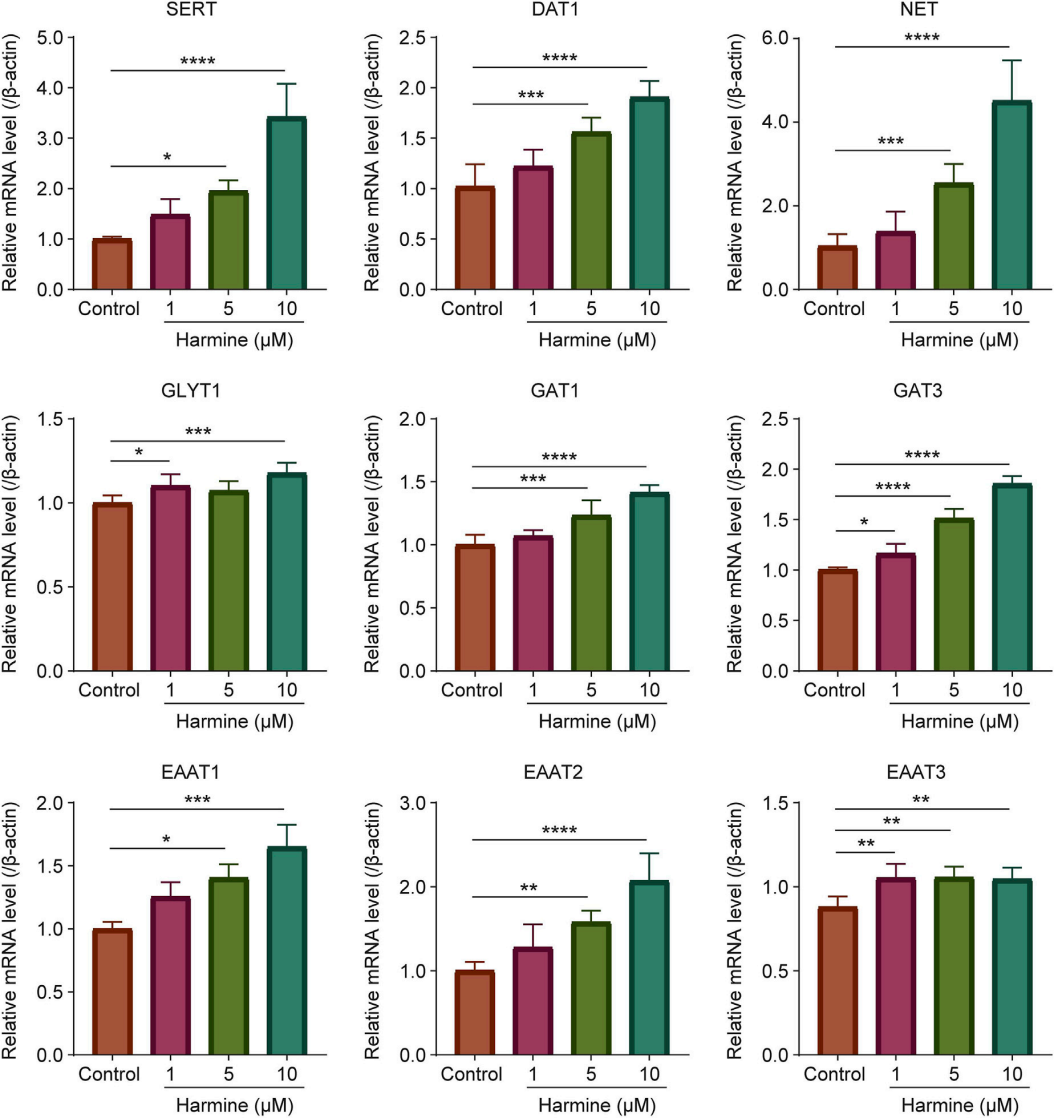
The effect of harmine on the mRNA expression of neurotransmitter transporters SERT, DAT1, NET, GLYT1, GAT1, GAT3, EAAT1, EAAT2 and EAAT3 in primary neurons. Data were presented as mean ± SD, n = 4–6/group. ^*^p < 0.05, ^**^p < 0.01, ^***^p < 0.001, ^****^p < 0.0001 compared with control group.

**FIGURE 6:**
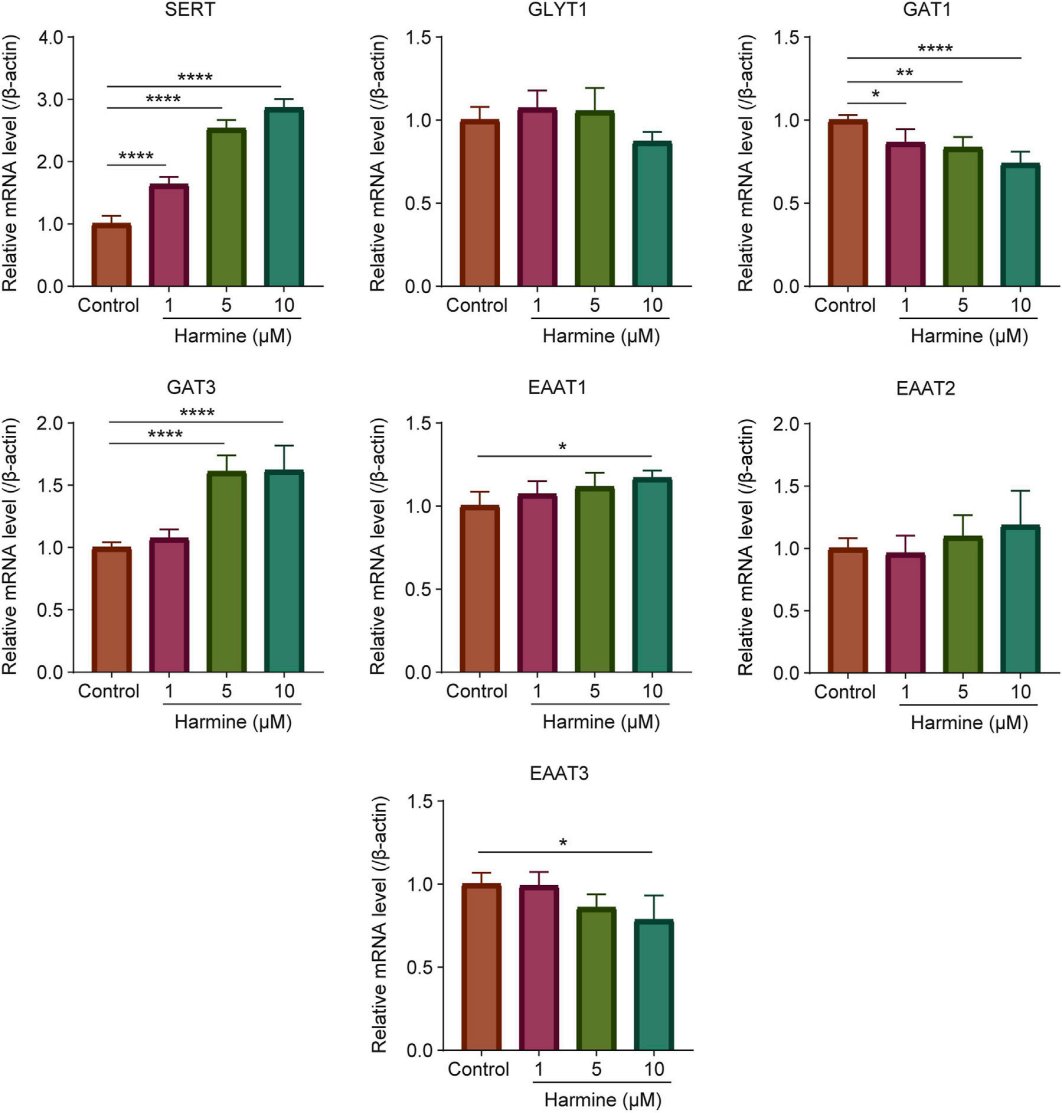
The effect of harmine on the mRNA expression of neurotransmitter transporters SERT, GLYT1, GAT1, GAT3, EAAT1, EAAT2 and EAAT3 in primary astrocytes. Data were presented as mean ± SD, n = 4–6/group. ^*^p < 0.05, ^**^p < 0.01, ^****^p < 0.0001 compared with control group.

To investigate whether harmine exerted a neuromodulatory effect, its impact on the expression of neurotransmitter transporters was also examined by quantitative real-time PCR. The results showed that harmine treatment significantly upregulated the mRNA expression of dopamine transporter 1 (DAT1), serotonin transporter (SERT), norepinephrine transporter (NET), gamma-aminobutyric acid (GABA) transporter 1 (GAT1), gamma-aminobutyric acid transporter 3 (GAT3), excitatory amino acid transporter 1 (EAAT1), excitatory amino acid transporter 2 (EAAT2), and excitatory amino acid transporter 3 (EAAT3) in neurons (Figure 5). In astrocytes, harmine significantly upregulated the mRNA expression of SERT and GAT3, while downregulated the mRNA expression of GAT1 (Figure 6). These results revealed that harmine significantly affected the mRNA expression of neurotransmitter reuptake transporters in both neurons and astrocytes, with a more pronounced effect observed in neurons. This might be attributed to differences in transporter expression levels between neurons and astrocytes, as well as variations in the underlying regulatory mechanisms involved. Notably, the impact was particularly evident on the transporters for serotonin, dopamine and norepinephrine.

### 3.4 Exploration and validation of receptors targeted by harmine

#### 3.4.1 Discovery of potential receptors

To further screen for receptors interacting with harmine using a human proteome microarray, harmine was first biotinylated to facilitate its detection and analysis. The characterization results of the harmine-biotin complex were shown in Supplementary Figure 5. The HPLC analysis showed that the purity of the purified product reached 98.65% (Supplementary Figure 5). The MS results, including the extracted ion chromatogram (XIC) and the ion fragments, were consistent with the predicted structure of the harmine-biotin complex (Supplementary Figure 6). NMR spectroscopy further confirmed that the structure of the purified product (harmine-biotin complex) was correct (Supplementary Figures 7–10). The ^1^H NMR data for the purified product were as follows: 1H NMR (400 MHz, DMSO) δ 12.24 (s, 1H), 8.35 (d, J = 8.6 Hz, 1H), 8.28–8.23 (m, 1H), 7.44–7.39 (m, 1H), 7.08 (dd, J = 8.6, 2.1 Hz, 1H), 6.50 (s, 1H), 6.41 (s, 1H), 4.33 (dd, J = 7.8, 5.0 Hz, 1H), 4.17 (ddd, J = 7.6, 4.3, 1.8 Hz, 1H), 3.21–3.12 (m, 1H), 2.88–2.80 (m, 1H), 2.66 (t, J = 7.3 Hz, 2H), 2.60 (d, J = 12.4 Hz, 1H), 1.71 (tt, J = 10.6, 6.1 Hz, 3H), 1.52 (ddq, J = 35.5, 14.7, 6.6 Hz, 2H). The ^13^C NMR data were as follows: 13C NMR (101 MHz, DMSO) δ 171.93, 162.81, 151.63, 142.05, 140.67, 133.91, 128.90, 123.45, 118.12, 115.15, 114.33, 105.31, 61.12, 59.26, 55.41, 33.46, 28.08, 28.04, 24.41, 18.57. These results demonstrated that the synthesized harmine-biotin complex met the requirements for human proteome microarray analysis and could be used to identify receptors that interact with harmine.

Finally, a total of 270 significantly specific proteins were detected filtering based on I Mean_Ratio≥1.4. Further screening focused on receptors related to neurotransmitter functions, particularly G protein-coupled receptors and ion channel receptors. Ultimately, two receptors, G protein-coupled receptor 85 (GPR85) and chloride intracellular channel 2 (CLIC2), were identified. The results of the human proteome microarray were shown in Table 3.

**TABLE 3:** The results of the human proteome microarray.

HAR-Bio	+	-	SNR
GPR85	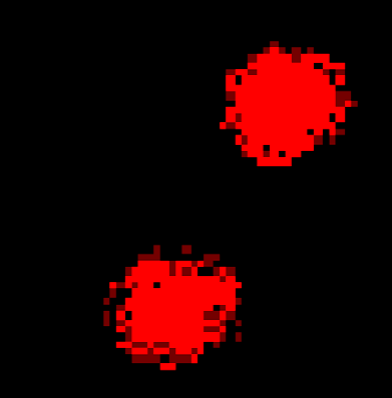	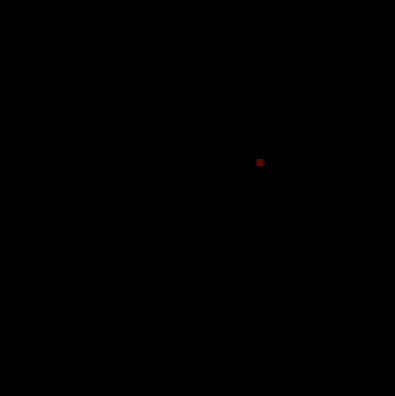	11.21
CLIC2	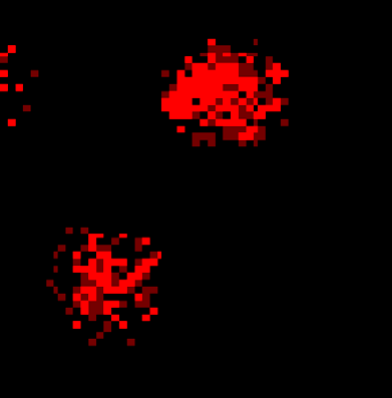	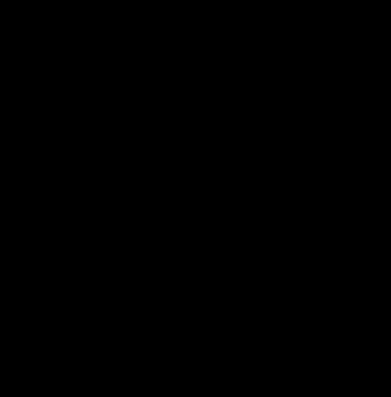	12.0

#### 3.4.2 Harmine exerted an inhibitory effect on the G protein-coupled receptor 85

**FIGURE 7:**
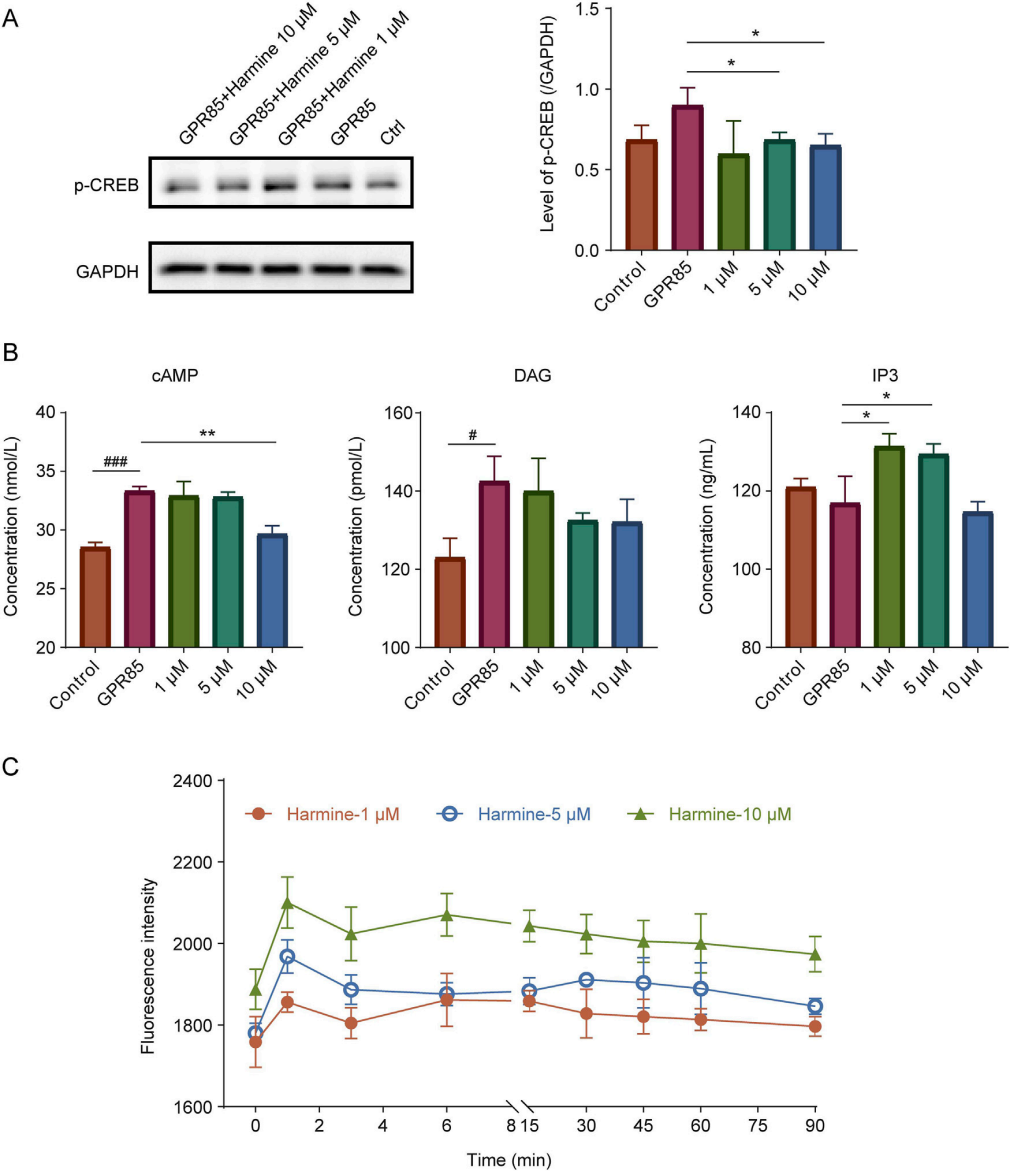
Validation of the interaction between harmine and its candidate receptors. **(A)** Western blotting analysis and quantification of p-CREB in GPR85-overexpressing cells following harmine treatment. **(B)** Measurement of second messenger levels (cAMP, DAG and IP3) in GPR85-overexpressing cells after harmine stimulation. **(C)** DiSBAC2(3) fluorescence intensity alterations in CLIC2-overexpressing cells after harmine stimulation. Data were presented as mean ± SD, n = 3/group. ^#^p < 0.05, ^###^p < 0.001 compared with control group; ^*^p < 0.05, ^**^p < 0.01 compared with GPR85 group.

To investigate the effect of harmine on the GPR85, the impact of harmine on the phosphorylation of CREB and on second messenger levels in cells were assessed. As shown in Figure 7A and Supplementary Figure 11, harmine treatment, particularly at 5 and 10 μM, significantly downregulated the expression of p-CREB (95% CI: −0.41 to −0.02 at 5 μM, −0.46 to −0.03 at 10 μM, p < 0.05). As shown in Figure 7B, harmine treatment at 10 μM significantly decreased cAMP levels (95% CI: −4.91 to −2.39, p < 0.01). Moreover, harmine treatment slightly decreased the DAG levels, while harmine treatment at 1 and 5 μM increased the IP3 levels (95% CI: 2.33–26.43 at 1 μM, 0.80–24.06 at 5 μM, p < 0.05). These results suggested that the effect of harmine on GPR85 might be inhibitory.

#### 3.4.3 Harmine modulated the membrane potential of SH-SY5Y cells

To further elucidate whether harmine could directly or indirectly modulate neuronal membrane potential and activity, its effect on membrane potential was investigated using DiSBAC2(3) fluorescence probe. After investigating different probe incubation time, it was found that the membrane potential differences were more pronounced across different concentrations of harmine, and the stability of the measurements was also better under the condition of 75 min incubation. Therefore, 75 min was selected as the optimal incubation time. As shown in Figure 7C, harmine treatment resulted in an increase in DiSBAC2(3) fluorescence intensity, with a more pronounced fluorescence change observed as the concentration of harmine increased. This finding suggested that harmine treatment induced depolarization in the cells.

## 4 Discussion

Harmine is an important endogenous compound and possesses various neuropharmacological effects. In this work, we attempted to investigate whether harmine exhibits properties of a neurotransmitter or neuromodulator. However, proving a compound as a novel neurotransmitter is challenging. Although harmine has been widely investigated for its pharmacological effects, studies specifically focused on its endogenous biosynthesis, specific transport mechanisms, and regulated release remain limited. Therefore, our research could only proceed according to the four essential criteria of a neurotransmitter to verify the role of harmine (Bian et al., 2023).

Inspired by the biosynthesis of β-CAs via P-S reaction catalyzed by strictosidine synthase (STR) in plants (Cao and Wang, 2021), we sought to identify mammalian proteins capable of catalyzing this reaction. Using bioinformatics approaches and techniques, we compared the STR base sequences from plants with the rat gene database to identify potential proteins with similar functions. Among them, the highest matching protein was APMAP-X1, which was selected for further activity verification. APMAP is widely distributed in mammals and has been demonstrated to be associated with various diseases, including gestational diabetes mellitus, polycystic ovary syndrome, primary Sjogren’s syndrome, demyelinating disease and Alzheimer’s disease (Ilhan et al., 2008; Gerber et al., 2019; Haley et al., 2020). Herein, APMAP was further demonstrated to catalyze the reaction between SEC and TRY to form strictosidine, confirming its P-S catalytic function. This result revealed that APMAP might serve as an important biotransformation enzyme mediating the P-S reaction in mammals.

Subsequently, based on the structure of harmine, MOT and AA were selected as substrates to verify the catalytic synthesis pathway of harmine *in vitro*. The results showed that purified APMAP-X1 effectively catalyzed the reaction between AA and MOT to form tetrahydroharmine, which was further oxidized to harmine by MPO. These findings preliminarily confirmed the existence of a corresponding biosynthetic pathway for harmine in mammals. However, given the absence of MOT in mammals, it remains unclear whether this pathway also occurs *in vivo*. Further studies are needed to identify the specific substrates and clarify the endogenous synthesis mechanism of harmine, and the possibility of alternative biosynthetic routes cannot be excluded.

To investigate the clearance and release pathways of harmine, we conducted an initial investigation on the metabolism, uptake, and release of harmine in synaptosomes. The results revealed that harmine could be metabolized within synaptosomes, taken up by synaptosomes, and released upon high K^+^ stimulation. Given that neurotransmitter reuptake primarily occurs at the presynaptic neuron terminals and astrocytes, we further performed experiments on primary neural cells. The results demonstrated that both neurons and astrocytes were capable of efficiently uptaking harmine, with maximal uptake achieved within 10 min. Additionally, the uptake of harmine by primary neural cells was dose-dependent. These findings suggested the presence of clearance pathways (involving metabolism and uptake) and release pathways for harmine in the synaptosomes, and harmine was capable of being cleared from the synaptic cleft. However, due to the lack of identified transporters mediating the uptake of harmine, it remains inconclusive whether harmine enters neurons via active transport or passive diffusion. Further studies are required to identify and characterize the specific transporter responsible for harmine uptake and to elucidate the mechanism underlying its release.

Moreover, harmine could also modulate the expression of the neurotransmitter transporters, which suggested that it might directly influence the uptake of neurotransmitters. Harmine might potentially enhance neurotransmitter reuptake and reduce neurotransmitter levels in the synaptic cleft by upregulating neurotransmitter transporters expression, thereby broadly modulating synaptic transmission and exerting multiple effects on neural functions. Li et al. (2011) found that harmine could increase EAAT2/GLT1 gene expression and glutamate uptake *in vivo*. In addition, tetrahydroharmine, the biosynthetic precursor of harmine, has been reported to inhibit 5-HT uptake in presynaptic neurons (Airaksinen et al., 1980). Considering the high degree of structural similarity between tetrahydroharmine and harmine, it is reasonable to hypothesize that harmine may exert a similar modulatory effect, although further experimental validation is required. This regulation effect may partly be attributed to harmine’s known inhibition of monoamine oxidase (MAO) activity. By inhibiting MAO, harmine may lead to elevated levels of monoaminergic neurotransmitters within neurons (Jiang et al., 2019). This increase could in turn activate compensatory feedback mechanisms, thereby altering the expression of neurotransmitter transporters. Harmine has also been shown to inhibit the activity of acetylcholinesterase (AChE) (Li et al., 2018; Jiang et al., 2019). Both enzymes are crucial for the metabolism of neurotransmitters. Furthermore, previous studies have demonstrated that harmine can regulate the neurotransmitter levels in memory-impaired mice and promote the formation of synaptic vesicles (Li et al., 2018; Xie et al., 2025). These findings implied that harmine played a crucial role in maintaining the dynamic balance of neurotransmitters and regulating transmission of neural signals, thereby influencing behavioral and cognitive processes. Collectively, these results further supported the potential of harmine as a neuromodulator.

β-Carboline alkaloids are a class of pharmacologically active compounds and are important in clinic treatment. They have a lot of pharmacological activities and possess the potential for treatment of various CNS diseases (Xie et al., 2021). Among them, harmine has been reported to inhibit MAO, AChE, and dual-specificity tyrosine-phosphorylation-regulated kinase 1A (DYRK1A), thereby exerting various pharmacological effects, including antitumor, antioxidant, anti-inflammatory, anti-Alzheimer’s disease and antidepressant effects (He et al., 2015; Jiang et al., 2019; Tarpley et al., 2021). In addition, previous studies have shown that harmine exhibits affinity for [^3^H]agonist-labeled 5-HT_2A_ receptor and 5-HT_2C_ receptor, but the functional roles of harmine at these sites remain unclear (Glennon et al., 2000). Brierley and Davidson (2013) found that harmine (300 nM) selectively enhanced dopamine (DA) efflux in the nucleus accumbens shell, an effect that could be attenuated by the 5-HT_(2A/2C)_ antagonist but not by the MAO inhibitor. Moreover, Liu et al. (2018) showed that harmine could enhance GABAergic transmission onto basal amygdala projection neurons, suggesting its potential role in modulating inhibitory synaptic activity. These findings suggested that harmine could exert its neuromodulatory effects through mechanisms beyond classical enzymatic inhibition. In this study, GPR85 and CLIC2 were identified as potential harmine-binding proteins that may be associated with its neurotransmitter-like properties. However, previously reported harmine-binding receptors were not detected in proteome microarray analysis. One possible reason is that the biotinylation site on harmine may interfere with its ability to interact with certain receptors, thereby masking potential binding events. This limitation may have restricted the comprehensive identification of harmine’s actual binding targets. GPR85, also known as SREB2 (Super Conserved Receptor Expressed in Brain 2), is an orphan G protein-coupled receptor highly conserved across species and predominantly expressed in the central nervous system, particularly in the hippocampus, cortex, and striatum (Matsumoto et al., 2005). GPR85 transcripts are predominantly detected in tissues of neuroectodermal origin, with high expression during early neuronal differentiation in the CNS, particularly in the developing cerebral cortex (Hellebrand et al., 2001). Current studies suggested that GPR85 is closely associated with psychiatric disorders and cognitive impairments. It functions as a negative regulator of neurogenesis and cognitive function (Chen et al., 2012; Alavi et al., 2018). Therefore, the inhibition of GPR85 may be a novel strategy to enhance hippocampal neurogenesis and improve cognitive abilities. In our study, harmine was found to bind with GPR85 and exhibited an inhibitory effect on GPR85. This result suggested that harmine may exert its therapeutic effects in improving psychiatric disorders and cognitive impairments by inhibiting GPR85, which is consistent with the known pharmacological properties of harmine. CLIC2, a member of the CLIC protein family, is expressed in various tissues including the brain, heart, and liver (Choudhury et al., 2022; Ozaki et al., 2022). Previous studies suggested that CLIC2 may regulate cardiac ryanodine receptors and modulate microglial function (Choudhury et al., 2022; Ozaki et al., 2022). However, its precise biological functions remain unclear. In this study, harmine was found to modulate the membrane potential in CLIC2-overexpressing cells and induce depolarization, suggesting a potential neuromodulatory effect. However, the underlying mechanisms remain unclear. Harmine may exert its effects through direct interaction with ion channels or indirectly by modulating other signaling pathways that ultimately lead to the changes of membrane potential. Further investigation is required to elucidate the precise signaling mechanisms of the two receptors and to determine how harmine exerts its effects through these targets.

When compared with classical neurotransmitters, harmine exhibited several shared features, including the presence in the synaptosomes, synaptic uptake and release, receptor binding, and involvement in neurotransmitter system regulation. However, differences are also apparent. Current study only demonstrated the presence of harmine in synaptosomes, however, it remains unclear whether harmine is localized within synaptic vesicles of presynaptic neurons. Unlike classical neurotransmitters that are transported by specific and well-characterized transporters, no dedicated transporter responsible for harmine uptake has yet been identified. Regarding receptor interactions, the effects of classical neurotransmitters following receptor binding have been well elucidated, whereas the downstream physiological effects resulting from harmine-receptor binding are still unclear. In addition, the classical mechanism of neurotransmitter release involves an influx of Ca^2+^ following neuronal stimulation, which subsequently triggers vesicle fusion and neurotransmitter release. However, whether the release of harmine from synaptosomes involves a similar Ca^2+^-dependent mechanism remains unclear (Mallik and Frank, 2023). Further investigations are needed to elucidate these aspects and the physiological effects of harmine at endogenous concentrations, which are essential for establishing harmine’s potential role as a neurotransmitter.

## 5 Conclusion

In conclusion, our research revealed the presence of two key enzymes in mammals, APMAP and MPO, which are essential for the endogenous synthesis of harmine. The presence of clearance pathways (involving metabolism and uptake) and release pathways for harmine within the synaptosomes was also confirmed. In addition, harmine could regulate the neurotransmitter-related enzyme activities (MPO, AChE, etc.) and reuptake transporters expression (SERT, DAT1, GAT1, GAT3, etc.) to exert its potential neuromodulatory effect. Importantly, harmine could inhibit GPR85 signaling and induce neuronal depolarization, further supporting its ability to influence neuronal excitability. Taken together, these findings provide preliminary evidence that harmine may exhibit neurotransmitter-like properties in certain aspects. However, direct evidence supporting harmine as a neurotransmitter is still limited. The current evidence more strongly supports the endogenous neuromodulatory role of harmine, while its role as a neurotransmitter requires further experimental validation to clarify the precise mechanisms by which harmine participates in neurotransmission.

## Data Availability

The original contributions presented in the study are included in the article/Supplementary Material, further inquiries can be directed to the corresponding author.
