# Supplementary material for: Exploration of the potential neurotransmitter or neuromodulator-like properties of harmine: evidence from synthesis to synaptic modulation
Source: Front Pharmacol. 2025 Jul 7;16:1588105. doi: 10.3389/fphar.2025.1588105 (PMC12277293; doi:10.3389/fphar.2025.1588105)
Supplement: Supplementary file 1 [file DataSheet1.pdf]

Supplementary Material

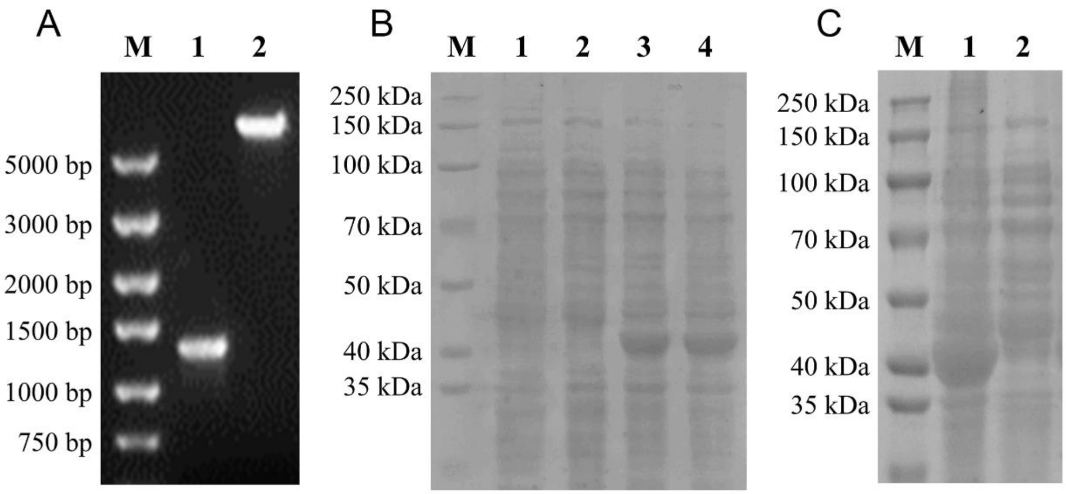

Supplementary Figure 1. Electrophoresis of DNA and protein. M: Marker. (A) Electrophoresis of PCR product and digested product. Channel 1: the APMAP-X1 PCR product, channel 2: the digested pET28(DE3) vector. (B) Electrophoresis of bacterial cells. Channel 1, 2: the uninduced cell, channel 3, 4: the induced cell. (C) Electrophoresis of supernatant and sediment. Channel 1: the pellet, channel 2: the supernatant.

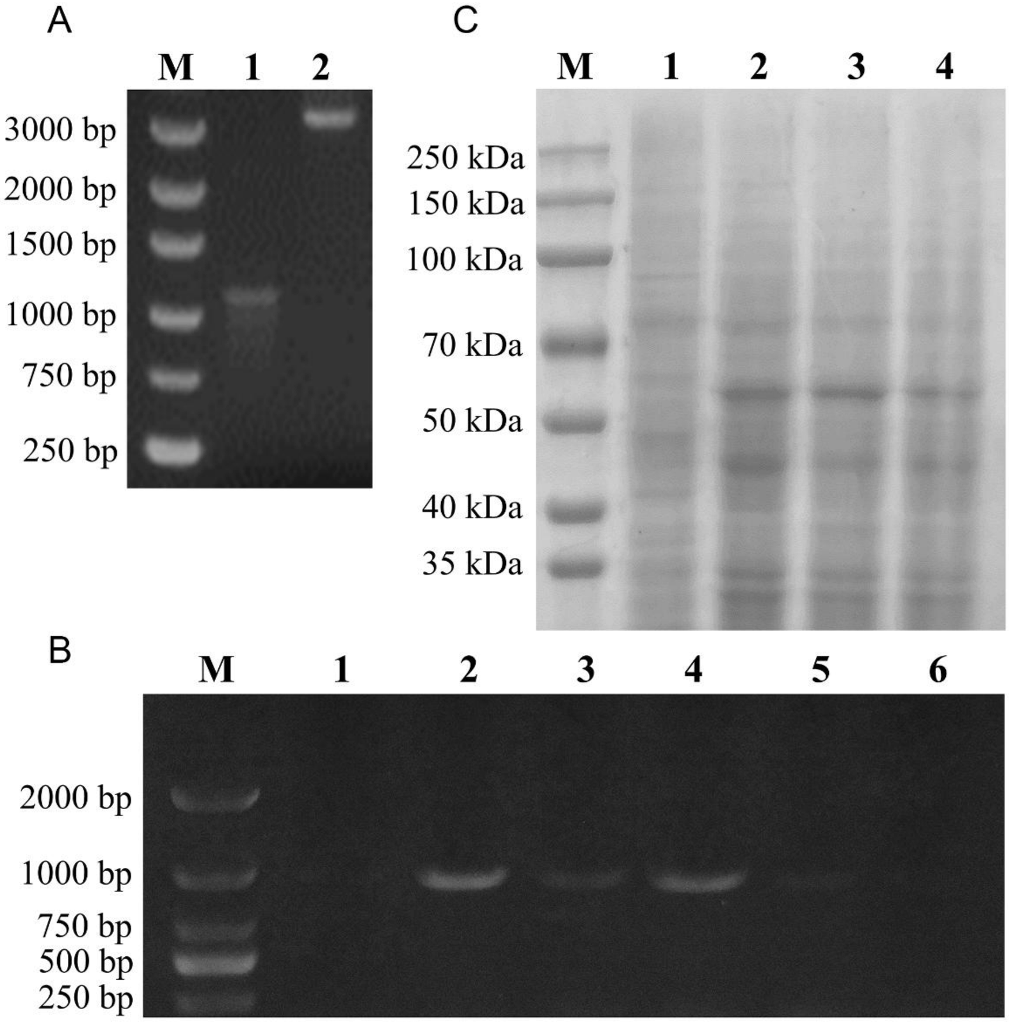

Supplementary Figure 2. Electrophoresis of DNA and protein. M: Marker. (A) Electrophoresis of PCR product and digested product. Channel 1: the APMAP-X1 PCR product, channel 2: the digested pGAPZB vector. (B) Results of agarose gel electrophoresis analysis. 1-5: PCR of APMAP-X1-PGAPZB transformed colonies, channel 6: PCR of pGAPZB transformed colonies. (C) Electrophoresis of GS115/pGAPZB and GS115/pGAPZB-APMAP-X1 protein. Channel 1: Insoluble protein in pGAPZB-GS115 colony, channels 2, 3, 4: Insoluble protein in APMAP-X1-PGAPZB-GS115 after 2, 4 and 6 days of induction.

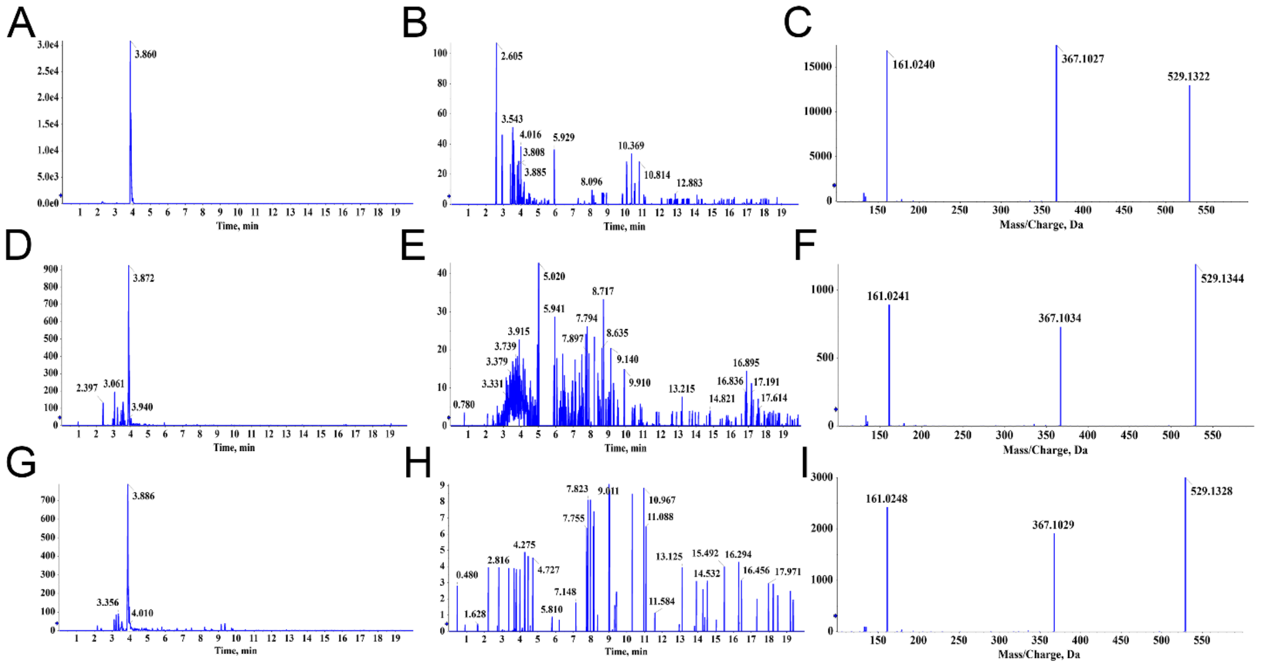

Supplementary Figure 3. Verification of catalytic activity of APMAP-X1 expressed in *Escherichia coli* and *Pichia pastoris*. The XIC (A) and secondary ion fragments (C) of strictosidine standard. The XIC (D) and secondary ion fragments (F) of incubation product catalyzed by APMAP-X1 expressed in *E. coli* BL21(DE3). The XIC (G) and secondary ion fragments (I) of incubation product catalyzed by APMAP-X1 expressed in *P. pastoris* GS115. (B) Negative control without APMAP-X1. (E) The XIC of incubation product without substrates (incubation without APMAP-X1 in *E. coli* BL21(DE3)). (H) Negative control (incubation without APMAP-X1 in *P. pastoris* GS115).

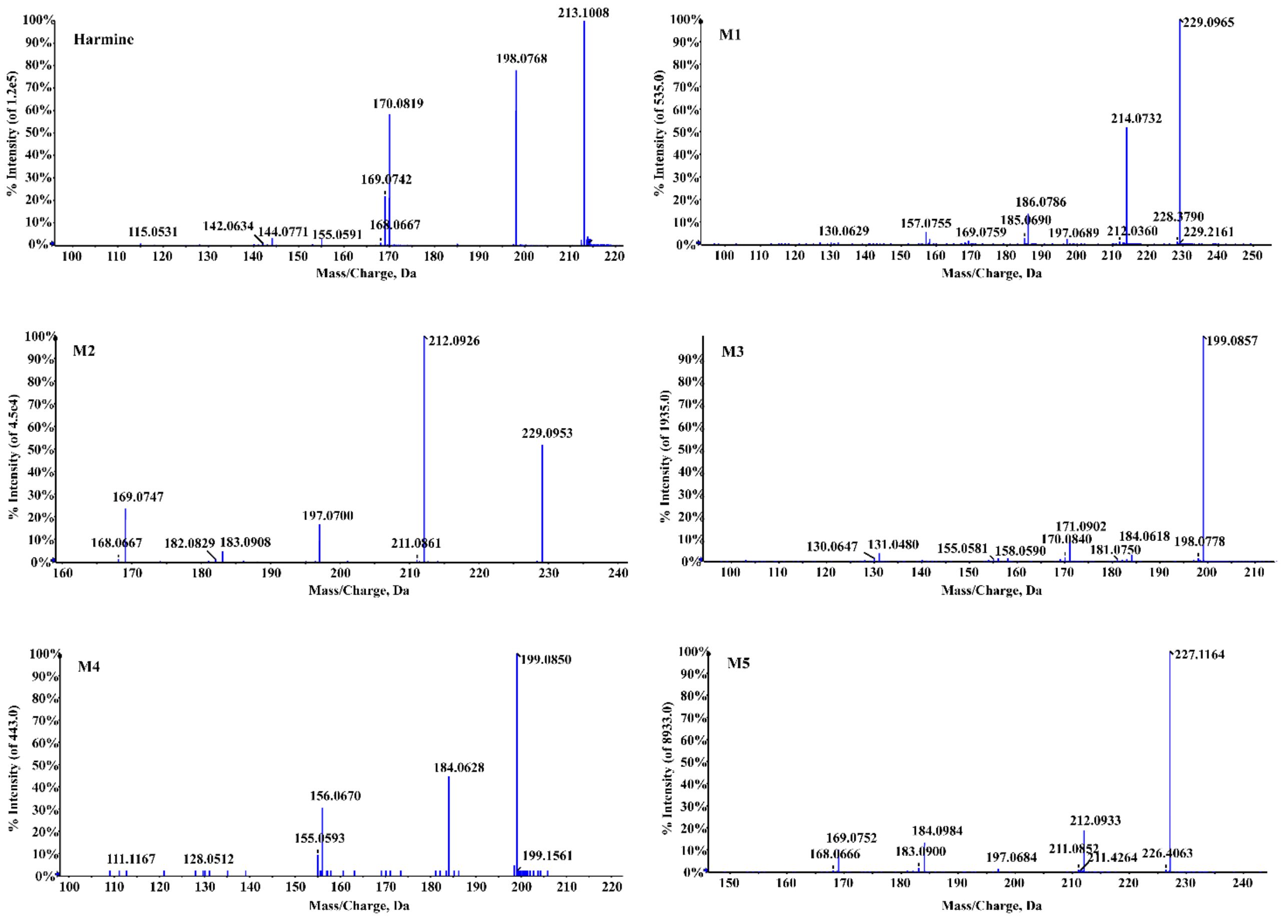

Supplementary Figure 4. The MS spectrum of harmine and metabolites.

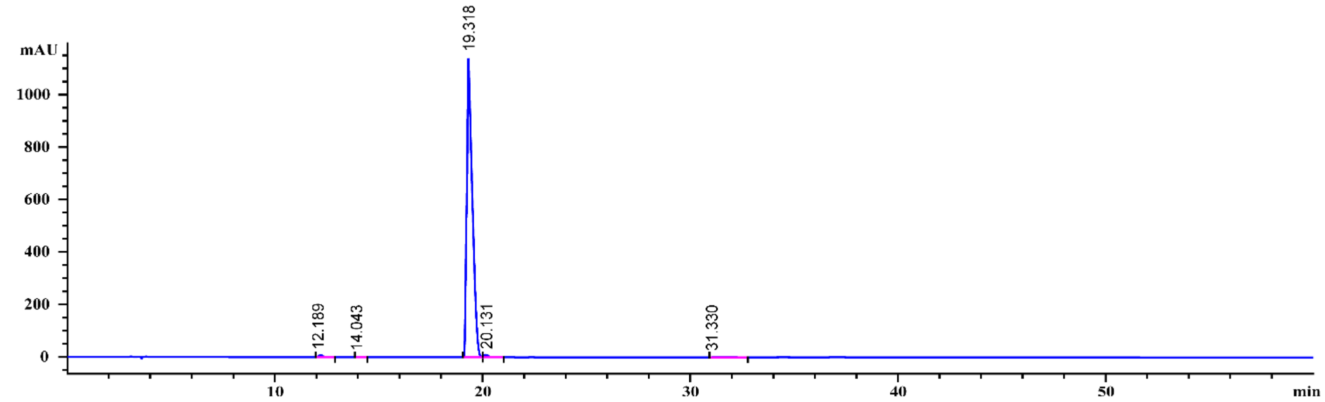

Supplementary Figure 5. The chromatogram of the purity assessment of harmine-biotin complex.

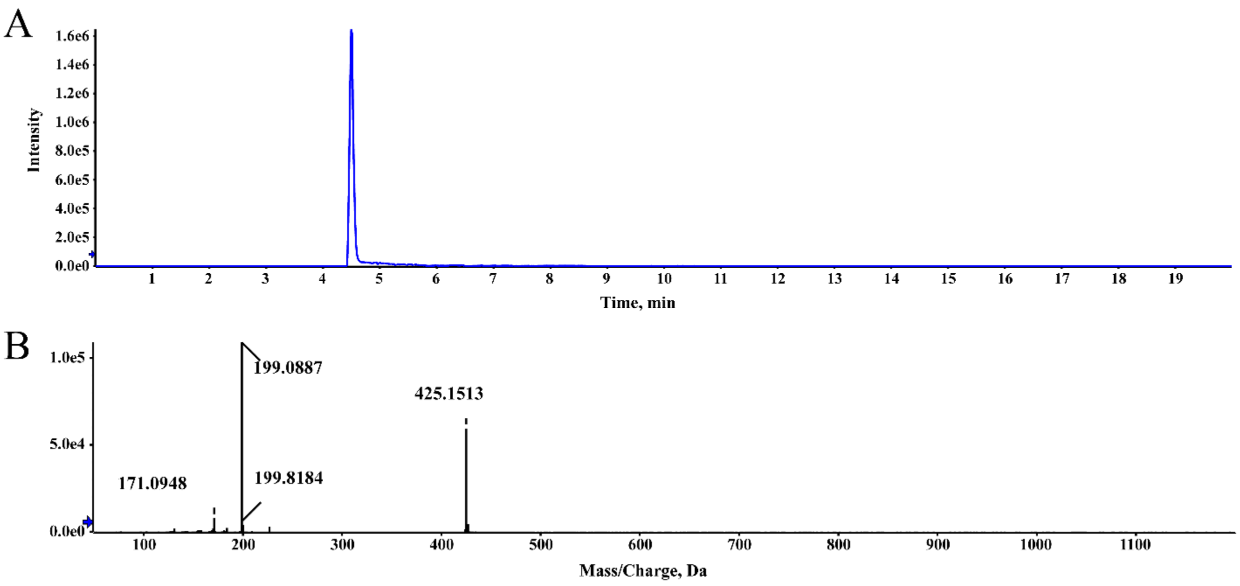

Supplementary Figure 6. The XIC (A) and secondary ion fragments (B) spectrums of harmine-biotin complex.

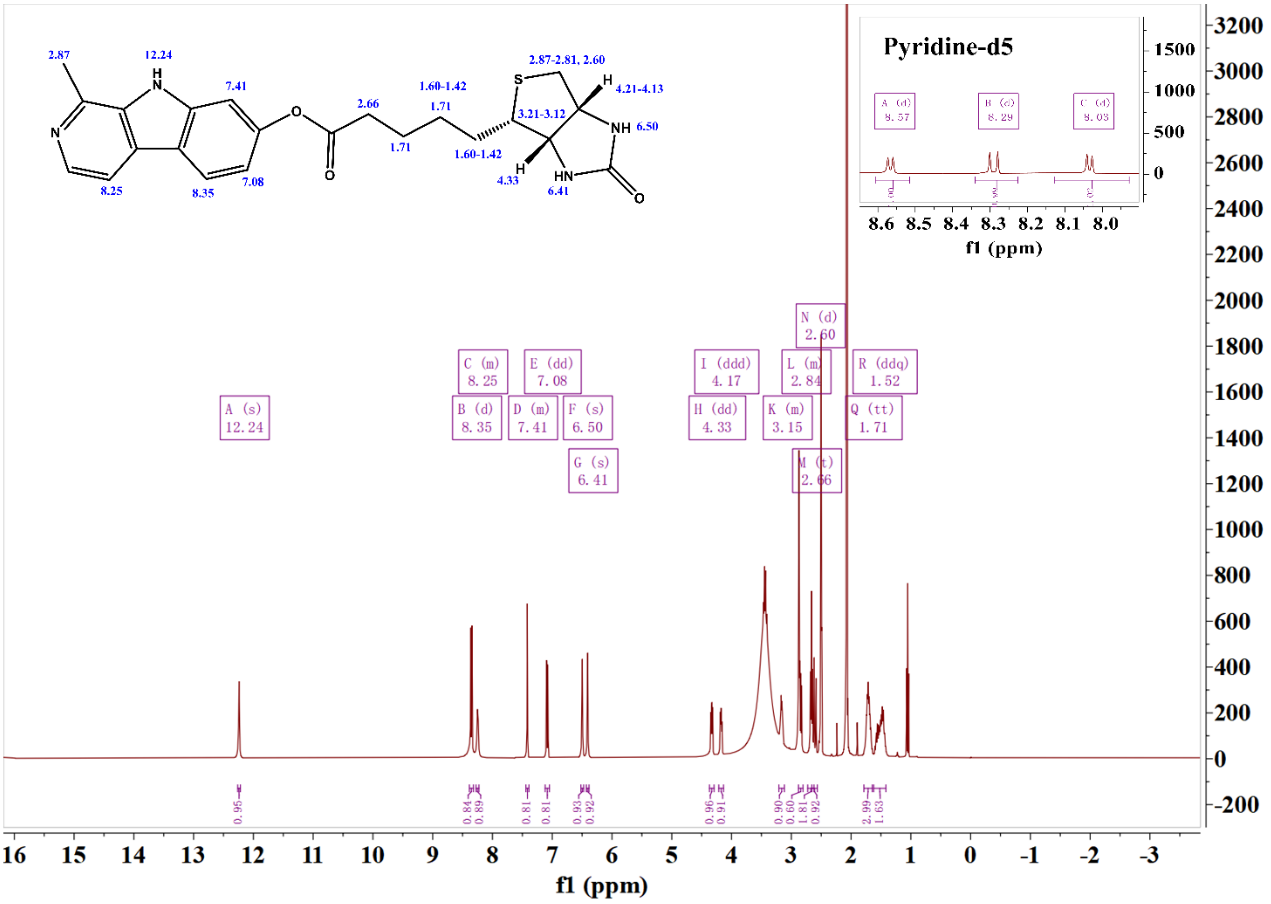

Supplementary Figure 7. The ^{1}H NMR spectrum of harmine-biotin complex.

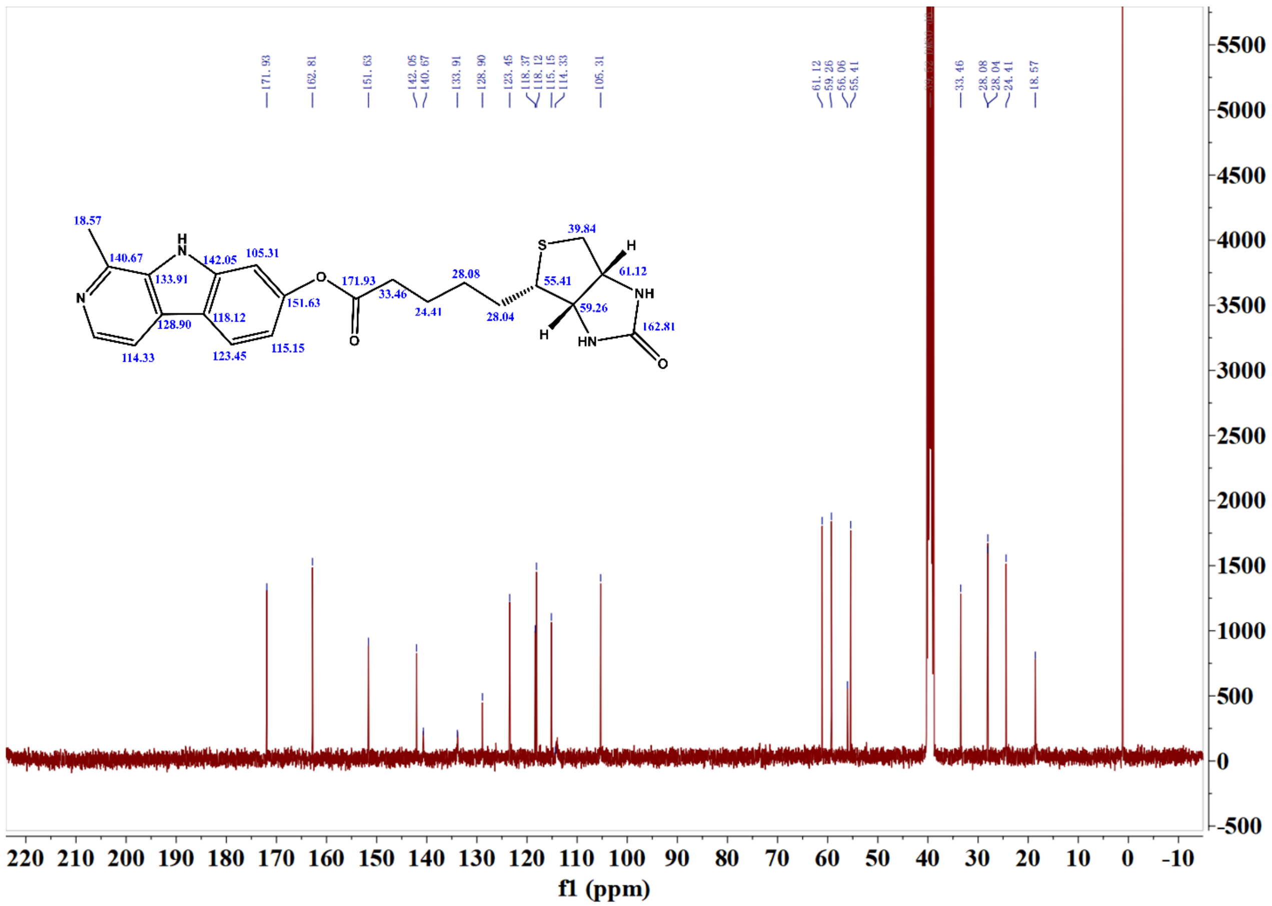

Supplementary Figure 8. The ^{13}C NMR spectrum of harmine-biotin complex.

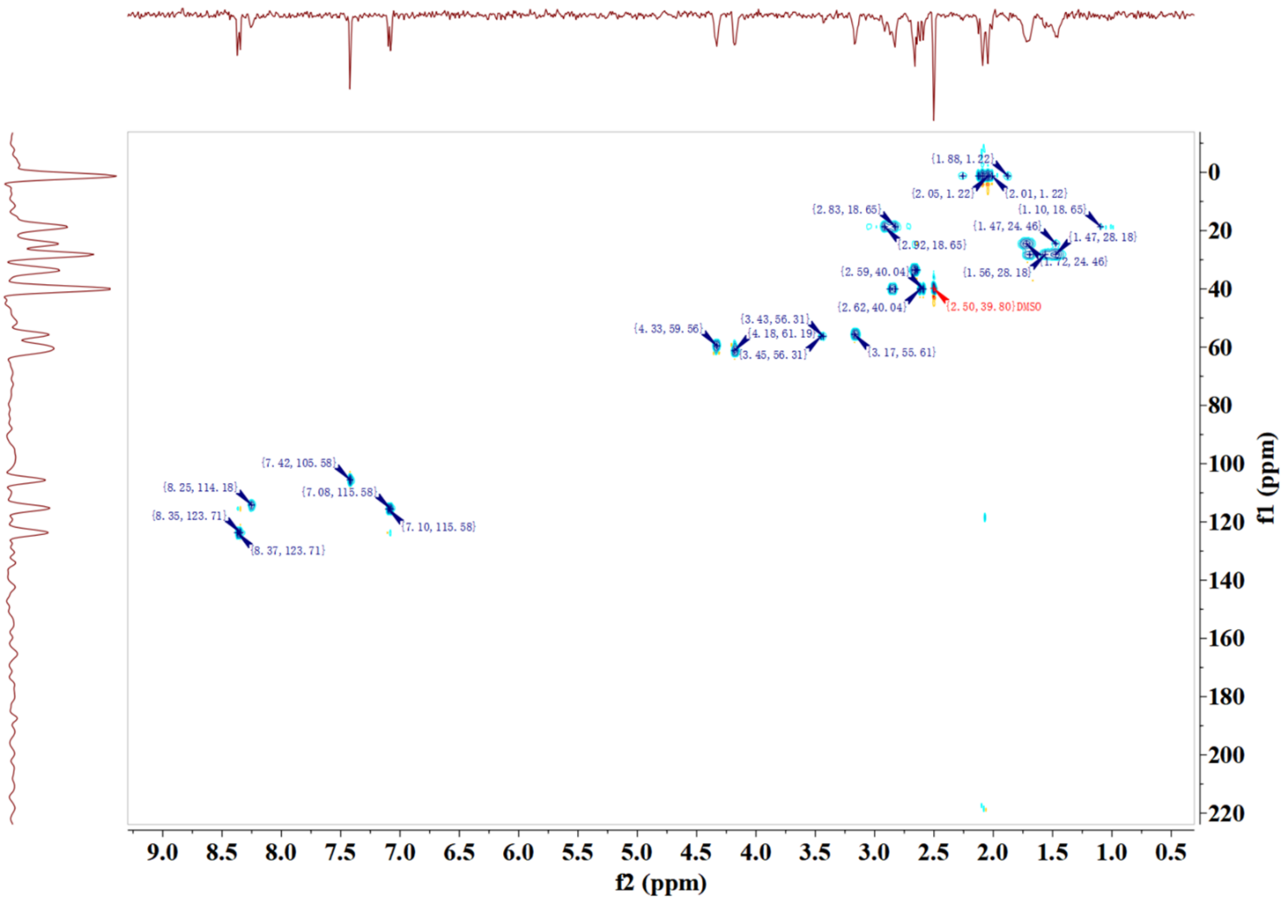

Supplementary Figure 9. The ^{1}H-^{13}C HSQC spectrum of harmine-biotin complex.

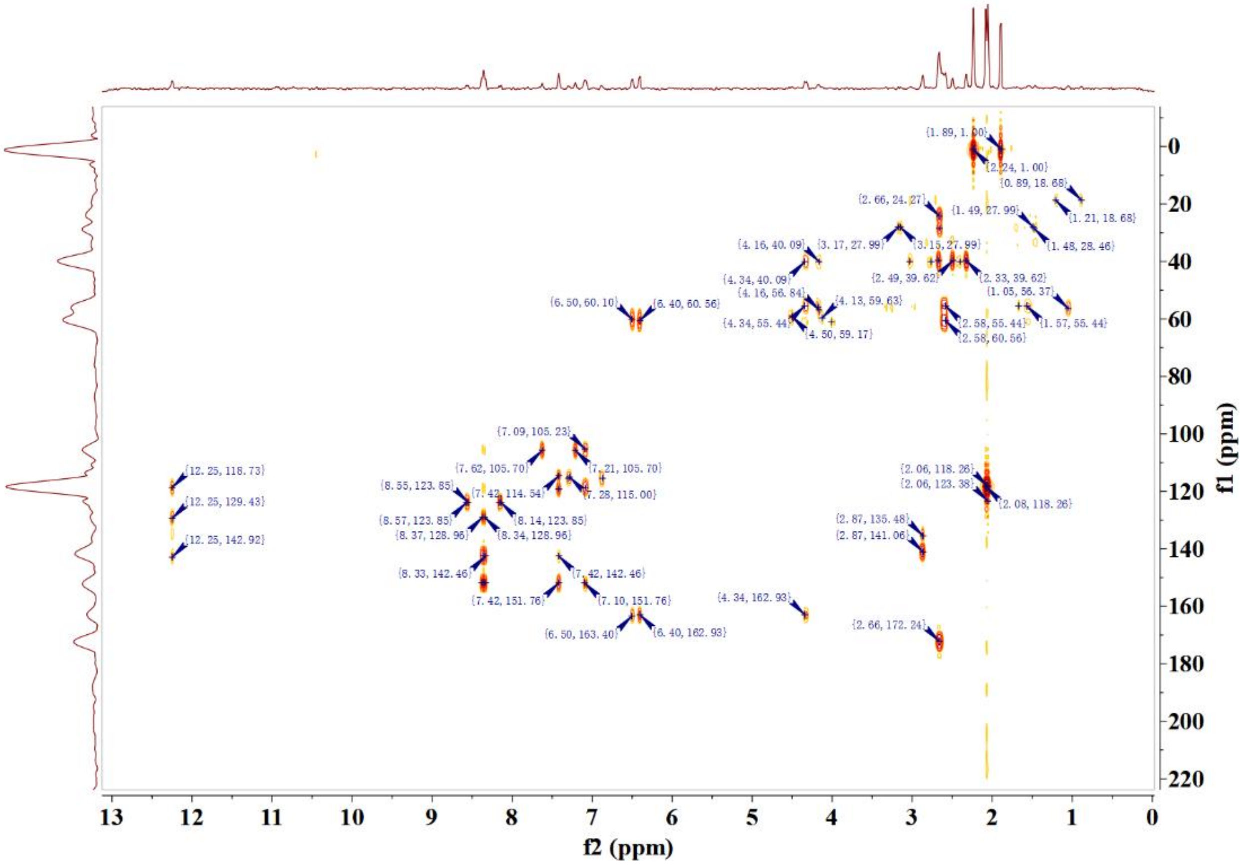

Supplementary Figure 10. The ^{1}H-^{13}C HMBC spectrum of harmine-biotin complex.

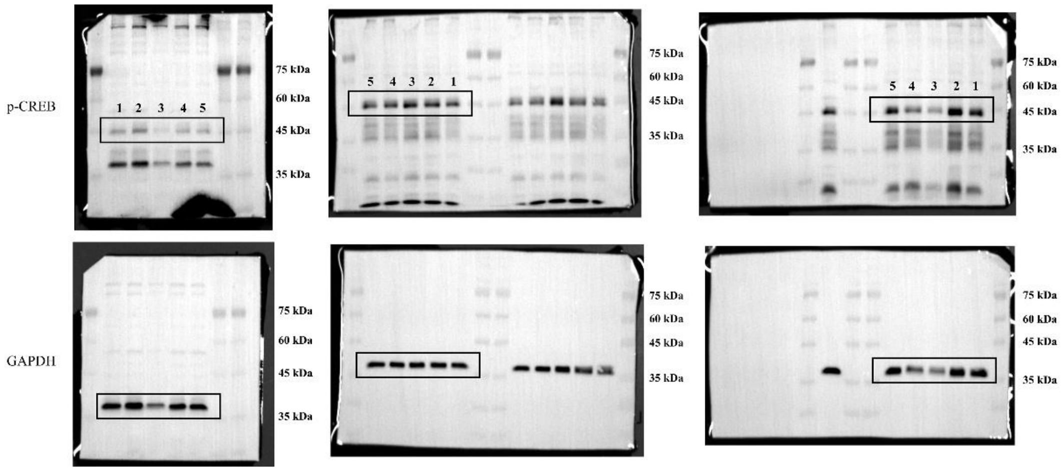

Supplementary Figure 11. Western blotting analysis of p-CREB in GPR85-overexpressing cells. 1: Ctrl, 2: GPR85, 3: GPR85+Harmine 1 μM, 4: GPR85+Harmine 5 μM, 5: GPR85+Harmine 10 μM.
